# Learning and Innovative Elements of Strategy Adoption Rules Expand Cooperative Network Topologies

**DOI:** 10.1371/journal.pone.0001917

**Published:** 2008-04-09

**Authors:** Shijun Wang, Máté S. Szalay, Changshui Zhang, Peter Csermely

**Affiliations:** 1 Department of Automation, Tsinghua University, Beijing, China; 2 Department of Medical Chemistry, Semmelweis University, Budapest, Hungary; University of East Piedmont, Italy

## Abstract

Cooperation plays a key role in the evolution of complex systems. However, the level of cooperation extensively varies with the topology of agent networks in the widely used models of repeated games. Here we show that cooperation remains rather stable by applying the reinforcement learning strategy adoption rule, Q-learning on a variety of random, regular, small-word, scale-free and modular network models in repeated, multi-agent Prisoner's Dilemma and Hawk-Dove games. Furthermore, we found that using the above model systems other long-term learning strategy adoption rules also promote cooperation, while introducing a low level of noise (as a model of innovation) to the strategy adoption rules makes the level of cooperation less dependent on the actual network topology. Our results demonstrate that long-term learning and random elements in the strategy adoption rules, when acting together, extend the range of network topologies enabling the development of cooperation at a wider range of costs and temptations. These results suggest that a balanced duo of learning and innovation may help to preserve cooperation during the re-organization of real-world networks, and may play a prominent role in the evolution of self-organizing, complex systems.

## Introduction

Cooperation is necessary for the emergence of complex, hierarchical systems [1]–[5]. Why is cooperation maintained, when there is a conflict between self-interest and the common good? A set of answers emphasized agent similarity, in terms of kin- or group-selection and compact network communities, which is helped by learning of successful strategies [2], [3]. On the other hand, agent diversity in terms of noise, variation of behavior and innovation, as well as the changing environment of the agent-community all promoted cooperation in different games and settings [3], [6]–[8].

Small-world, scale-free or modular network models, which all give a chance to develop the complexity of similar, yet diverse agent-neighborhoods, provide a good starting point for the modeling of the complexity of cooperative behavior in real-world networks [9]–[13]. However, the actual level of cooperation in various games, such as the Prisoner's Dilemma or Hawk-Dove games is very sensitive to the topology of the agent network model [14–16, Electronic supplementary material S1 – ESM1 – Table S1.1]. In our work we applied a set of widely used network models and examined the stability of cooperation after repeated games using the reinforcement learning strategy adoption rule, Q-learning. To examine the surprising stability of cooperation observed, when using Q-learning, we approximated the complex rules of Q-learning by designing a long-term versions of the best-takes-over and other strategy adoption rules as well as introducing a low level of randomness to these rules. We found that none of these features alone results in a similar stability of cooperation in various network models. However, when applied together, long-term (‘learning’) and random (‘innovative’) elements of strategy adoption rules can make cooperation relatively stable under various conditions in a large number of network models. Our results have a wide application in various complex systems of biology from the cellular level to social networks and ecosystems.

## Results

### Sensitivity of cooperation on network topology

**Figure 1:**
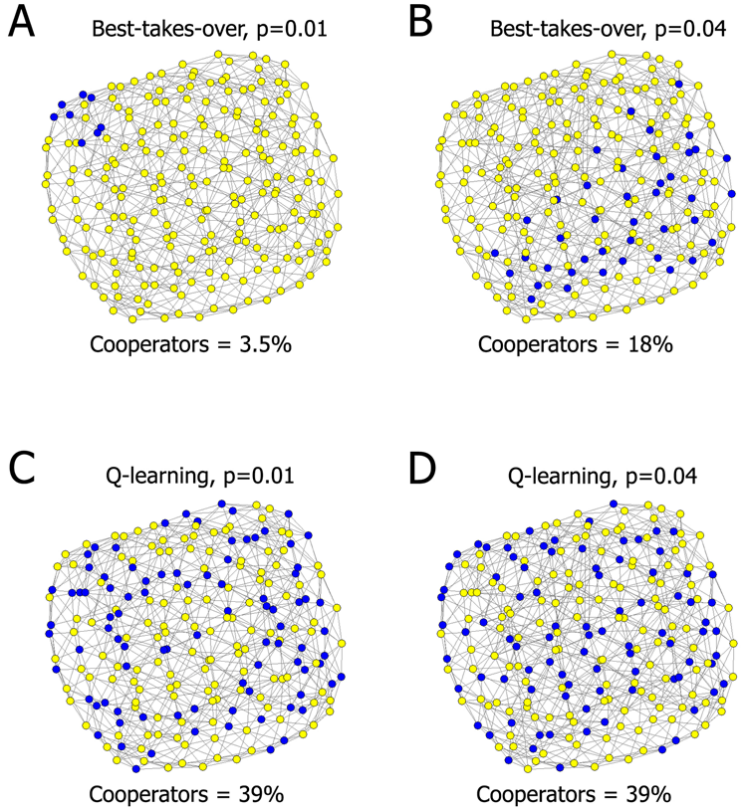
A long-term learning adoption rule, Q-learning improves and stabilizes cooperation of agents forming various small-world networks in Prisoner’s Dilemma games. The modified Watts-Strogatz small-world network was built on a 15×15 lattice, where each node was connected to its eight nearest neighbors. The rewiring probabilities of the links placed originally on a regular lattice were 0.01 (left panels) and 0.04 (right panels), respectively. For the description of the canonical repeated Prisoner's Dilemma game, as well as the best-takes-over (top panels) and Q-learning (bottom panels) strategy adoption rules see Methods and the ESM1. The temptation level, T was 3.6. Networks showing the last round of 5,000 plays were visualized using the Kamada-Kawai algorithm of the Pajek program [46]. Dark blue dots and diamonds correspond to cooperators and defectors, respectively. The Figure shows that both the extent and distribution of cooperators vary, when using the best-takes-over strategy adoption rule (see top panels), while they are rather stable with the Q-learning strategy update rule (see bottom panels).

As an illustrative example for the sensitivity of cooperation on network topology, we show cooperating agents after the last round of a ‘repeated canonical Prisoner's Dilemma game’ (PD-game) on two, almost identical versions of a modified Watts-Strogatz-type small-world model network [13], [17]. Comparison of the top panels of Figure 1 shows that a minor change of network topology (replacement of 37 links from 900 links total) completely changed both the level and topology of cooperating agents playing with a best-takes-over short term strategy adoption rule. We have observed a similar topological sensitivity of cooperation in all combinations of (a) other short-term strategy adoption rules; (b) a large number of other network topologies; (c) other games, such as the extended Prisoner's Dilemma or Hawk-Dove games (ESM1 Figures S1.1 and S1.6).

### Q-learning stabilizes cooperation in different network topologies

On the contrary to the general sensitivity of cooperation to the topology of agent-networks in PD-games using the short-term strategy adoption rule shown above, when the long-term, reinforcement learning strategy adoption rule, Q-learning was applied, the level and configuration of cooperating agents showed a surprising stability (cf. the bottom panels of Figure 1). Just oppositely to the short-term strategy adoption rule shown on the top panels of Figure 1, the Q-learning strategy adoption rule (a) is based on the long-term experiences of the agents from all previous rounds allowing some agents to choose a cooperative strategy despite of the current adverse effects, and (b) is an ‘innovative’ strategy adoption rule [3] re-introducing cooperation even under conditions, when it has already been wiped out from the network-community completely [18], [19].

**Figure 2:**
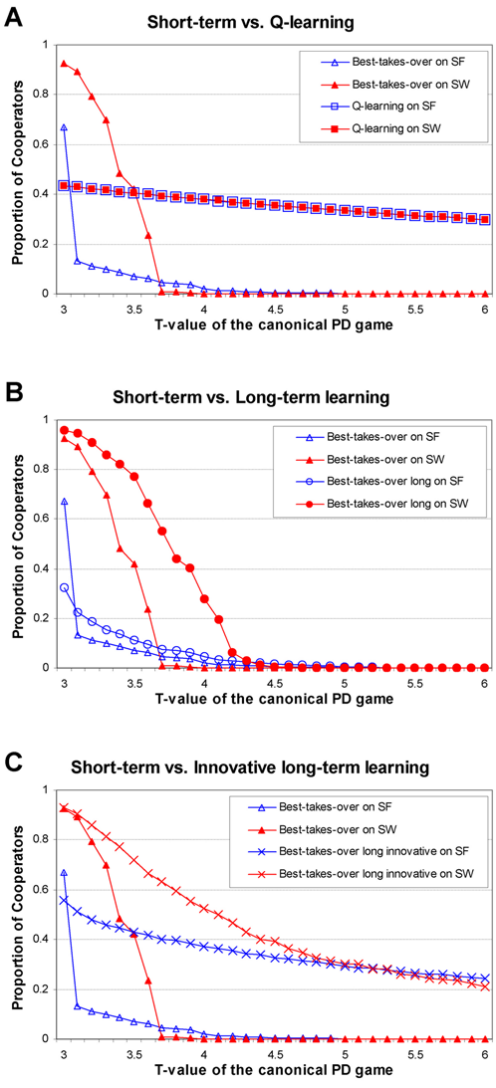
Long-term learning elements of strategy update rules help, while a low level of randomness relatively stabilizes cooperation in Prisoner's Dilemma games played on various networks. Small-world (SW, filled, red symbols) networks were built as described in the legend of Figure 1. The Barabasi-Albert-type scale-free networks (SF, open, blue symbols) contained 2,500 nodes, where at each construction step a new node was added with 3 new links attached to the existing nodes. For the description of the canonical repeated Prisoner's Dilemma game, as well as that of the best-takes-over (triangles, all panels), the Q-learning (rectangles, top panel) the best-takes-over long (circles, middle panel), and the best-takes-over long innovative (crosses, P_innovation_ = 0.0002, bottom panel) strategy adoption rules, see Methods and the ESM1. For each strategy adoption rules and *T* temptation values 100 random runs of 5,000 time steps were executed. The figure shows that long-term, ‘learning-type’ elements of strategy update rules help cooperation in Prisoner's Dilemma games played on various networks. A low level of randomness (also called as ‘innovation’ in this paper) brings the level of cooperation closer in different network topologies.

Extending the observations shown on Figure 1 we decided to compare the level of cooperation in PD-games on small-world and scale-free networks at various levels of temptations (T, the defector's payoff, when it meets a cooperator) in detail. The top panel of Figure 2 shows that the cooperation level of agents using the best-takes-over strategy adoption rule rapidly decreased with a gradual increase of their temptation to defect. This was generally true for both small-world, and scale-free networks leaving a negligible amount of cooperation at T-values higher than 4.5. However, at smaller temptation levels the level of cooperation greatly differed in the two network topologies. Initially, the small-world network was preferred, while at temptation values higher than 3.7, agents of the scale-free network developed a larger cooperation. The behavior of agents using the Q-learning strategy adoption rule was remarkably different (top panel of Figure 2). Their cooperation level remained relatively stable even at extremely large temptation values. Moreover, the cooperation levels of agents using Q-learning had no significant difference, if we compared small-world and scale-free networks. This behavior continued at temptation values higher than 6 (data not shown). We have observed the same differences in both the extent of cooperation at extremely high temptations (or gains of hawks meeting a dove in the Hawk-Dove game) and the topological sensitivity of cooperation in all combinations of (a) other short-term strategy adoption rules; (b) a large number of other network topologies; (c) other games, such as the extended Prisoner's Dilemma or Hawk-Dove games (ESM1 Figures S1.2 and S1.6).

### Long-term strategy adoption rules improve but do not stabilize cooperation in different networks

Next we wanted to see, if other long-term strategies besides Q-learning can also promote cooperation between agents. In Q-learning agents consider a long-term experience learned in all the past rounds of the play. Therefore, we modified the best-takes-over strategy adoption rule allowing the agents to use accumulative rewards of their neighbors in all past rounds instead of the reward received just in the last round. In agreement with our expectations, both on small-world and scale-free networks this long-term strategy adoption rule outperformed its short-term version allowing a larger number of agents to cooperate – especially at high temptation values. Importantly, the differences between cooperation levels observed in small-world and scale-free networks were even greater, when we applied the long-term strategy adoption rule compared to its short-term version (middle panel of Figure 2). We have received very similar results in all combinations of (a) other short- and long-term strategy adoption rule pairs; (b) a large number of other network topologies; (c) other games, such as the extended Prisoner's Dilemma or Hawk-Dove games. Long-term learning strategy adoption rules also promoted cooperation (albeit at lower efficiency than in case of complex network structures), when we used networks re-randomized after each play, or randomly picked agents (ESM1 Figures S1.3–S1.6). As a summary, we conclude that long-term strategy adoption rules (‘learning’ instead of simple imitation) allow a larger cooperation, but do not stabilize the cooperation-fluctuations inflicted by the different topologies of the underlying networks, which leaves the remarkable topological stability of the Q-learning strategy adoption rule still unexplained.

### Low level of randomness of the strategy adoption rules is needed to stabilize cooperation level in different network topologies

Next we tested, if the innovative elements of the Q-learning strategy adoption rule may contribute to the stability of cooperation in various network topologies. For this, we constructed an ‘innovative’ version of the long-term version of the best-takes-over, ‘non-innovative’ strategy adoption rule by adding a low level of randomness instructing agents to follow the opposite of the selected neighbor's strategy with a pre-set *P_innovation_* probability (see Methods). Cooperation levels achieved by the innovative long-term best-takes-over strategy adoption rule are shown on the bottom panel of Figure 2. At temptation values smaller than T = 3.8 the innovative long-term version of the best-takes over strategy adoption rule outperformed Q-learning, which resulted in a larger proportion of cooperating agents (cf. top and bottom panels of Figure 2). However, at high temptation values Q-learning proved to be more efficient in maintaining cooperation. Most importantly, cooperation levels in small-world and scale-free networks were much closer to each other, when using the long-term innovative strategy-adoption rule, than either the ‘only long-term’, or short-term versions of the same strategy adoption rule (Figure 2). At high temptation values cooperation levels of long-term innovative strategy adoption rules on small-world and scale-free networks were converging to each other and even to the cooperation level observed, when using the Q-learning strategy adoption rule. We have received very similar results in combinations of (a) other innovative short- and long-term strategy adoption rules; (b) a large number of other network topologies; (c) other games, such as the extended Prisoner's Dilemma or Hawk-Dove games (ESM1 Figures S1.7 and S1.8). According to the expectations [8], the stabilizing role of the randomness in the strategy adoption rules depended on the actual value of the pre-set *P_innovation_* probability, and showed an optimum at intermediary *P_innovation_* levels, where the actual value of optimal *P_innovation_* depended on the strategy adoption rule and network topology. The effect of changes in *P_innovation_* was much more pronounced in case of scale-free networks than at small-world networks, which is a rather plausible outcome, since the larger irregularity of scale-free networks makes the re-introduction of extinct strategies a lot more crucial (ESM1 Figure S1.8).

**Figure 3:**
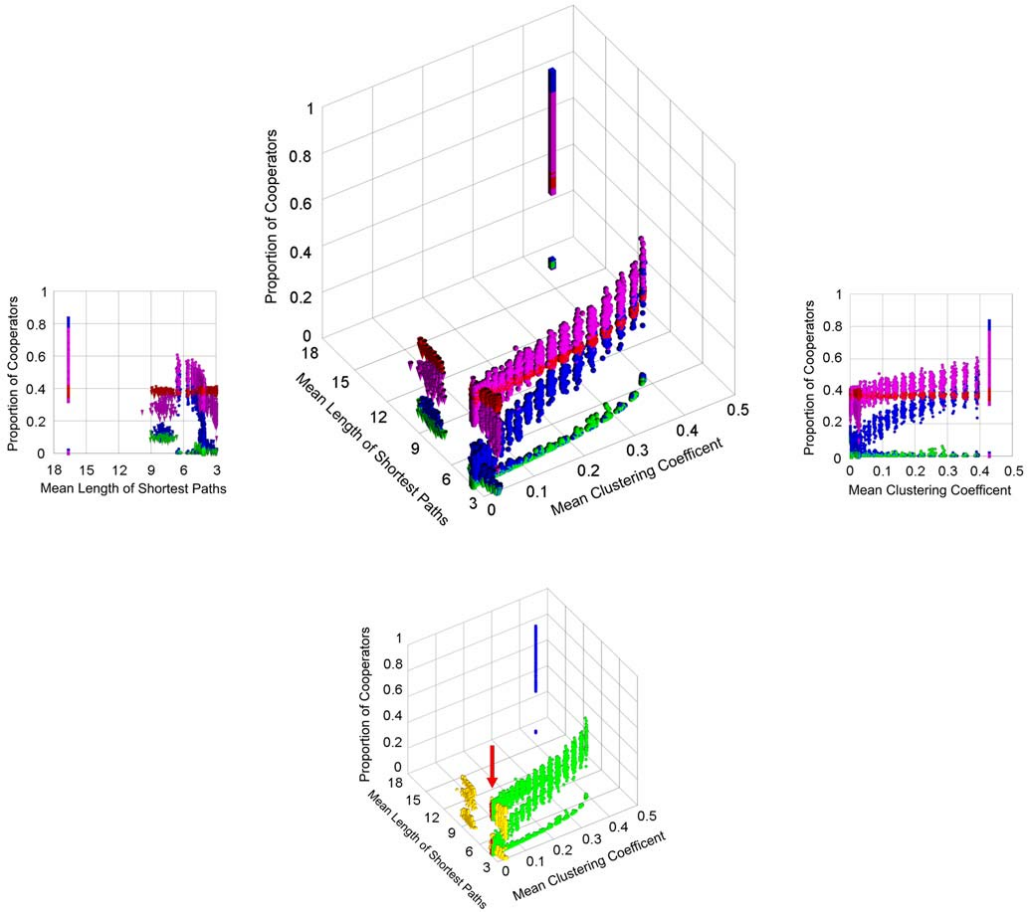
Long-term learning and innovative elements of strategy adoption rules, when applied together allow cooperation in a large number of model networks. (Top middle panel) The small-world (spheres) and scale-free (cones) model networks were built as described in the legends of Figures 1 and 2. The rewiring probability, *p* of the links of the original regular lattices giving small-world networks was increased from 0 to 1 with 0.05 increments, the number of edges linking each new node to former nodes in scale-free networks was varied from 1 to 7, and the means of shortest path-lengths and clustering coefficients were calculated for each network. Cubes and cylinders denote regular (p = 0) and random (p = 1.0) extremes of the small-world networks, respectively. For the description of the canonical repeated Prisoner's Dilemma game, as well as the best-takes-over (green symbols); long-term learning best-takes-over (blue symbols); long-term learning innovative best-takes-over (magenta symbols) and Q-learning (red symbols) strategy adoption rules used, see Methods and the ESM1. For each network 100 random runs of 5,000 time steps were executed at a fixed *T* value of 3.5. (Left and right panels) 2D side views of the 3D top middle panel showing the proportion of cooperators as the function of the mean length of shortest paths or the mean clustering coefficient, respectively. (Bottom middle panel) Color-coded illustration of the various network topologies used on the top middle panel. Here the same simulations are shown as on the top middle panel with a different color-code emphasizing the different network topologies. The various networks are represented by the following colors: regular networks – blue; small-world networks – green; scale-free networks – yellow; random networks – red (from the angle of the figure the random networks are behind some of the small-world networks and, therefore are highlighted with a red arrow to make there identification easier). The top middle panel and its side views show that the best-takes-over strategy adoption rule (green symbols) at this high temptation level results in a zero (or close-to-zero) cooperation. As opposed to this, the long-term best-takes-over strategy adoption rule (blue symbols) raise the level of cooperation significantly above zero, but the individual values vary greatly at the different network topologies. When the long-term strategy adoption rule is combined with a low level of randomness (magenta symbols) the cooperation level stays in most cases uniformly and its variation becomes high greatly diminished. Q-learning stabilizes cooperation further even at regular networks, which otherwise give an extremely variable outcome.

We have shown so far that long-term, learning strategy adoption rules help the development of cooperation, while ‘innovative’ strategy adoption rules make the cooperation level more independent from the actual network topology. Figure 3 illustrates how the cooperative network topologies were expanded, when we used long-term learning and ‘innovative’ versions of the best-takes-over strategy adoption rule as well as Q-learning at a high level of temptation, which made cooperation especially difficult. The application of the best-takes-over strategy adoption rule resulted in non-zero cooperation only sporadically. Cooperation levels using the long-term best-takes-over strategy adoption rule varied greatly, and still had several network configurations with zero cooperation. On the contrary, the two ‘innovative’ long-term learning strategy adoption rules had a much higher than zero cooperation in almost all networks tested, and the cooperation level remained fairly stable using a great variety of network topologies. This was especially true for Q-learning, which gave a stable level of cooperation even at regular networks (Figure 3), which result in a high instability of cooperation (see ESM1 Table S1.1). We have received very similar results in extended Prisoner's Dilemma and Hawk-Dove games (ESM1 Figures S1.9 and S1.10).

## Discussion

As a summary, our simulations showed that long-term learning strategy adoption rules promote cooperation, while innovative elements make the appearance of cooperation less dependent from the actual network topology in two different games using a large number of network topologies in model networks. We must emphasize that the term ‘learning’ is used in our paper in the sense of the collection and use of information enriching and diversifying game strategy and behavior, and not in the restricted sense of imitation, or directed information-flow from a dominant source (the teacher) pauperizing the diversity of game strategies. The help of learning in promoting cooperation is already implicitly involved in the folk theorem, which opens the theoretical possibility for the emergence of cooperation at infinitely repeated games [3], [20]. Learning, communication, negotiation, reputation-building mechanisms have all been shown to promote cooperation in various simulations as well as in games with groups of a variety of living organisms, including animals and humans (ESM1 Table S1.2). With the current work we have extended these findings showing that agents can markedly improve their cooperation, when they are allowed to consider long-term experiences either of their own (Q-learning) or their neighbors (other long-term strategies used), and this ‘shadow of the past’ [21] acts similarly at a great variety of network topologies.

We use the term ‘innovation’ in the sense of irregularities in the selection of adoption rules of game strategy. Therefore, ‘innovation’ may be caused by errors, mutations, mistakes, noise, randomness and temperature besides the *bona fide* innovation of conscious, intelligent agents. Our term, ‘innovation’ allows the change of the strategy adoption rules, therefore allows (increases) the evolvability [22] of our model system. Innovative strategies help to avoid ‘herding’, when agents start to use a uniform strategy and behavior forming synchronous clusters (ESM1 Figures S1.11, S1.12 and data not shown). Innovation increases game diversity and complexity, which, similarly to the stabilizing effect of weak links in a large variety of static networks, may significantly stabilize network dynamics (probably by helping the convergence of possible outcomes; [23]). Irregularities in network topology, noise, stochastic resonance, stochastic focusing and innovative strategies were shown to promote cooperation in various simulations as well as in games of primates and humans (ESM1 Table S1.3). However, the innovation-driven relative stabilization of cooperation in various network topologies is a novel finding reported here.

Cooperation helps the development of complex network structures [4], [5], [24]. Network dynamics and evolution lead to a large variety of link re-arrangements [25], [26]. Network evolution is full of stochastic ‘errors’, and often results in the development of a higher average degree [25], which makes cooperation more difficult [15], [16]. The highly similar cooperation levels of scale-free networks with different average degrees and of many other network topologies of model networks (Figure 3, ESM1 Figures S1.9 and S1.10) show that innovative long-term learning strategy adoption rules may provide a buffering safety-net to avoid the deleterious consequences of possible overshoots and errors in network development on cooperation. Our simulations showed (Figure 2, ESM1 Figures S1.2 and S1.6) that the help of innovative long-term learning is especially pronounced at conditions, where the relative cost of cooperation is the highest making cooperation most sensitive to the anomalies of network evolution [15]. This extreme situation is more easily reached, when the whole system becomes resource-poor, which makes all relative costs higher. Resource-poor networks develop a set of topological phase transitions in the direction of random → scale-free → star → fully connected subgraph topologies [27]. This further substantiates the importance of our findings that long-term, innovative learning allows a larger ‘cooperation-compatible’ window of these topologies, thus helps to avoid the decomposition of network structure in case of decreasing system resources due to e.g. an environmental stress. Further work is needed to show the validity of our findings in real-world networks as well as in combination with network evolution.

Our current work can be extended in a number of ways. The complexity of the game-sets and network topologies offers a great opportunity for a detailed equilibrium-analysis, similarly to that described by Goyal and Vega-Redondo [28]. The cited study [28] allows a choice of the interacting partners (an option denied in our model), which leads to another rich field of possible extensions, where the network topology is changing (evolving) during the games such as in the paper of Holme and Ghoshal [29]. Similarly, a detailed analysis of link rearrangement-induced perturbations, avalanches like in the paper of Ebel and Bornholdt [30] as well as exploration of a number of other topological re-arrangements would also significantly extend the current results. Such topology-changes may include

- hub-rewiring including the formation and resolution of ‘rich-clubs’, where hub-hub contacts are preferentially formed [31], [32];
- emergence of modularity beyond to our data in ESM1 Figure S1.4;
- appearance and disappearance of bridge-elements between modules;
- changes of modular overlaps and module hierarchy, etc.

Tan [33] showed that cooperation helps faster learning. This, when combined with our current findings may lead to a self-amplifying cycle between cooperation and learning, where cooperation-induced learning promotes cooperation. Emerging cooperation alleviates a major obstacle to reach a higher level of network hierarchy and complexity [4]. In social networks learning establishes trust, empathy, reputation and embeddedness [34]–[37], and the benefits of learning by multiple generations are exemplified by the development of traditions, norms and laws. These give the members of the society further reasons for withholding their individual selfishness, thereby reaching a higher network complexity and stability. We believe that learning and innovation (in forms of repeated, interaction-driven, or random network remodeling steps, respectively or using the Baldwin-effect, see ESM1 Discussion) help the evolution of cooperation between agents other than human beings or animals, including proteins, cells or ecosystems [23], [38], and were crucial in the development of multi-level, self-organizing, complex systems.

## Methods

### 

#### Games

In both the Hawk-Dove and the Prisoner's Dilemma games, each agent had two choices: to cooperate or to defect. In the repeated, multi-agent Hawk-Dove game the benefit of defectors is higher than that of cooperators, when they are at low abundance, but falls below cooperator benefit, when defectors reach a critical abundance [12], [13]. On the contrary, in the Prisoner's Dilemma game defection always has a fitness advantage over cooperation. The canonical parameter-set of the Prisoner's Dilemma game (*R* = 3, *P* = 1, *S* = 0, the *T*, temptation value varies between 3 to 6; 3 is not included; where R is the reward for mutual cooperation, P is the punishment for mutual defection, S and T are the payoffs for the cooperator and defector, respectively, when meeting each other) restricts cooperation more, than the parameter set of the extended (also called as ‘weak’) Prisoner's Dilemma game (*R* = 1, *P* = 0, *S* = 0 with *T* values ranging from 1 to 2; [11]–[13]). (When we tried the parameter set of *R* = 1, *P* = 0.2, *S* = 0.1 with T values ranging from 1.0 to 2.0, we have received very similar results; data not shown.)

In the Hawk-Dove games (or in the conceptually identical Snowdrift and Chicken games [13], [39], [40]) each agent had two choices: to defect (to be a hawk) or to cooperate (to be a dove). When a hawk met a dove, the hawk gained *G* benefits, whereas the payoff for the dove was zero. Two hawks suffered a (*G*−*C*)/2 cost each upon encounter, where *C*>*G* was the cost of their fight. When two doves met, the benefit for each dove was *G*/2. If not otherwise stated, the cost of injury (C, when a hawk met a hawk) was set to 1. The value of G varied from 0 to 1 with the increments of 0.1. If we want to compare the above, usually applied nomenclature of the Hawk-Dove games with that of the Prisoner's Dilemma games, R = G/2, P = (G−C)/2, S = 0 and T = G.

In Hawk-Dove games T>R>S>P, in the extended (also called ‘weak’) Prisoner's Dilemma game T≥R>P≥S, while in the canonical Prisoner's Dilemma game T>R>P>S. This makes the following order of games from less to more stringent general conditions allowing less and less cooperation: Hawk-Dove game>extended Prisoner's Dilemma game>canonical Prisoner's Dilemma game. Due to this general order, we showed the results of the canonical Prisoner's Dilemma game in the main text, and inserted the results of the two other games to the Electronic Supplementary Material S1 (ESMS1).

In our simulations each node in the network was an agent, and the agent could interact only with its direct neighbors. Agents remained at the same position throughout all rounds of the repeated games, and they were neither exchanged, nor allowed to migrate. If not otherwise stated, games started with an equal number of randomly mixed defectors and cooperators (hawks and doves in the Hawk-Dove game), and were run for 5,000 rounds (time steps). The payoff for each agent in each round of play was the average of the payoffs it received by playing with all its neighbors in the current round. In our long-term learning strategy adoption rules introduced below, the accumulative payoff means the accumulation of the average payoffs an agent gets in each round of play. Average payoff smoothes out possible differences in the degrees of agents, and in several aspects may simulate real-world situations better than non-averaged payoff, since in real-world situations agents usually have to observe a cost of maintaining a contact with their neighbors [39]–[41]. Moreover, average payoff helps the convergence of cooperation levels as the rounds of the game (time steps) proceed, what we indeed observed in most of the cases (with a few exceptions noted in the text), and helps to avoid ‘late-conversions’ occurring mostly in scale-free networks after 10,000 or more time steps using non-averaged payoffs. With this method it was enough to calculate the proportion of cooperators as the average ratio of cooperators of the last 10 rounds of the game (if not otherwise stated) for 100 independent runs.

#### Strategy adoption rules

In Prisoner's Dilemma and Hawk-Dove games our agents followed three imitation-type, short-term strategy adoption rules, the ‘pair-wise comparison dynamics’ (also called as ‘replicator dynamics’), ‘proportional updating’ and ‘best-takes-over’ (also called as ‘imitation of the best’) strategy adoption rules [13]. We call these rules strategy adoption rules and not evolution rules to avoid the mis-interpretation of our games as cellular automata-type games, where agents are replaced time-to-time. In our games no replacement took place, therefore these games were not evolutionary games in this strict sense. All strategy adoption rules had synchronous update, meaning that in each round of play the update took place after each agent had played with all their neighbors. To avoid the expansion of parameters with the differential placements of various agents in complex network structures all agents used the same strategy adoption rule in the agent-network. In the three strategy adoption rules we applied initially (‘best-takes-over’, ‘pair-wise comparison dynamics’ and ‘proportional updating’) all agents were myopic, and made their decisions based on the average payoffs gained in the previous round.

#### Pair-wise comparison dynamics strategy adoption rule

In the ‘pair-wise comparison dynamics’ strategy adoption rule [13] for any agent *i*, a neighboring agent *j* was selected randomly, and agent *i* used the strategy of agent *j* with a probability of *p_i_*. In our experiments the probability was determined as
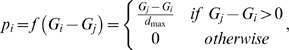
where *d*_max_ = (*G*+*C*)/2 (for Hawk-Dove games) or *d*_max_ = max(*T*, *R*) (for Prisoner's Dilemma games), which was the largest gap of gain between two agents in one round of play. *G_i_* and *G_j_* were the average payoffs received by agent *i* and *j* respectively in the current round of play.

#### Proportional updating strategy adoption rule

For the ‘proportional updating’ strategy adoption rule [13] agent *i* and all its neighbors competed for the strategy of agent *i* with the probability *p_i_*, which was determined as 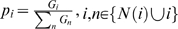 where *N*(*i*) was the neighborhood of agent *i* and *G_i_* was the average payoff received by agent *i* in the current round of play. Since *p* is a probability, *C* was added to each *G_i_* to avoid negative values. For Prisoner's Dilemma games, because the reward for an agent is always greater than or equal to zero, there was no need to increase the value of *G_i_*.

#### Best-takes-over strategy adoption rule

In the ‘best-takes-over’ strategy adoption rule (also called as imitation of the best strategy adoption rule, [13]) agent *i* adopted the strategy of that agent selected from *i* and its neighbors, who had the highest average payoff in the last round of play.

#### Q-learning strategy adoption rule

As a reinforcement learning [19] strategy adoption rule, we used Q-learning [18], where agents learned an optimal strategy maximizing their total discounted expected reward in the repeated game. In Q-learning we assumed that the environment constituted a discrete Markov process with finite states. An agent chose action *a_t_* from a finite collection of actions at time step, *t*. The state of the environment changed from state *s_t_* to *s_t_*_+1_ after the action of the agent, and the agent received the reward *r_t_* at the same time. The probability of state transition from *s_t_* to *s_t_*_+1_ when the agent chose action *a_t_* was
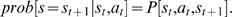



The task of the agent was to learn the optimal strategy to maximize the total discounted expected reward. The discounted reward meant that the rewards received by the agent in the future were worth less than that received in the current round. Under a policy π denoting how the agent selected the action at its actual state and reward, the value of state, *s_t_* was
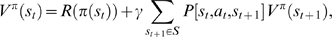
where *R*(π(*s_t_*)) is the expected reward of state *s_t_* under policy π and γ(0<γ<1) is the discount factor.

The theory of Dynamic Programming [19] guarantees that there is at least one optimal stationary policy, π^*^, which can be written as
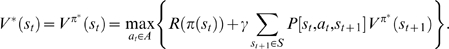



The task of Q-learning was to learn the optimal policy, π, when the initial conditions of both the reward function and transition probabilities were unknown. If the environment model (reward model and transition probabilities of states) is known, then the above problem can be solved by using Dynamic Programming. Watkins and Dayan [18] introduced Q-learning as incremental Dynamic Programming. The idea of Q-learning is to optimize a Q-function, which can be calculated iteratively without the estimate of environment model. For this having a policy, π, we defined the Q-value as:
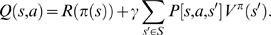
Q-learning consisted of a sequence of distinct stages or episodes. The Q value of state-action pair (*s_t_*, *a_t_*) can be learned through the following iterative method:
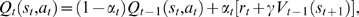
where 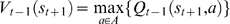 and α*_t_* controls the learning and convergence speed of Q-learning.

In repeated multi-agent games, the state of each agent was affected by the states of its direct neighbors. Those neighbors constituted the environment of the agent. The reward of the agent *i* after taking action *a_t_*(*i*) was defined as:
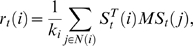
where *M* was the payoff matrix, *S_t_*(*i*) was a column vector indicating the state of agent *i* at round *t*, *k_i_* was the number of neighbors of agent *i* and *N*(*i*) was the set contains all the direct neighbors of agent *i*. The values of elements of *S_t_*(*i*) were 0 or 1 and 1 indicated that agent *i* was in the corresponding state. In such a repeated multi-agent game, Q-learning meant that each agent tried to optimize its total discounted expected reward in the repeated game. The optimal strategy was approximated by an iterative annealing process. For this for each agent, the selection probability (Boltzmann-probability) of action *a_i_* at time step *t* was defined as
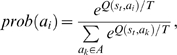
where *T* was the annealing temperature. In our experiments we selected the discount factor, γ*_t_* = 0.5, since in the initial experiments we found that this value is helpful to achieve high levels of cooperation. The initial annealing temperature was set to 100 in Hawk-Dove and extended Prisoner's Dilemma games, while it was raised to 10,000 in canonical Prisoner's Dilemma games to extend the annealing process [42]. In all cases the annealing temperature was decreased gradually by being divided by *t* in each round of the game till it reached a low bound of 0.001. In order to control the convergence speed of Q-learning, α = 1/(1+*TimesVisited*(*s*, *a*)) where *TimesVisited*(*s*, *a*) was the number of times that the state-action pair (*s*, *a*) had been visited at time step *t*. In this way α decreased gradually with the time.

#### Long-term learning and innovative strategy adoption rules

Long-term learning strategy adoption rules were generated by considering the accumulative average payoffs instead of instantaneous average rewards in the update progress during each round of play for all strategy adoption rules used. In both short term and long-term innovative strategy adoption rules, agent *i* used the opposite strategy of the selected neighbor (for proportional updating and best-takes-over strategy adoption rules, the neighborhood included agent *i* itself) in the last round of play with probability of *P_innovation_*, which was 0.0001 in case of Hawk-Dove and extended Prisoner's Dilemma games, while 0.0002 in case of canonical Prisoner's Dilemma games, if not otherwise stated (like in the legend of ESM1 Figure S1.8). In innovative strategy adoption rules agent *i* adopted the strategy of the selected neighbor with a probability of 1−*P_innovation_*.

#### Network construction

In our work we used a set of widely adopted model networks to simulate the complexity of real-world situations. Generation of the Watts-Strogatz-type small-world model network [17] was modified according to Tomassini *et al.* [13] to avoid the heterogeneity in node degrees, which arose during the Watts-Strogatz-type rewiring process changing the regular lattice to a small-world network. Such heterogeneity was shown to have a rather big influence on the level of cooperation [13], [43]. At the generation of the Barabasi-Albert-type scale-free network [44], we started from an initial fully connected graph of ‘m’ nodes (where ‘m’ ranged from 1 to 7), and added the new nodes with ‘m’ novel links as specified at the individual Figure legends. In the modular networks described by Girvan and Newman [45] each network had a scale-free degree distribution, contained 128 nodes, and was divided into 4 communities. The average degree was 16. Modularity (community structure) was gradually decreased at ‘levels’ 1, 5, 10 and 16, where ‘level 1’ meant that for each node in the network, the expected number of links between a node and the nodes which were in other communities was 1 (e.g. low compared to the average degree of 16). With increasing ‘level’ the community structure gradually decreased.

#### Network visualization

At the visualization the coordinates of the small-world networks with a rewiring probability of p = 0.01 were used for the p = 0.04 networks to avoid the individual variations of the Pajek-figures [46] and to help direct comparison. With 15×15 agents the final representations of cooperators showed a moderate variability. This was almost negligible, when 50×50 agents were used (data not shown). However, 15×15 agents gave a better visual image than the crowded, bulky 50×50 version. Therefore, we opted to include this variant to Figure 1. We have selected those figures from the results of 15×15 agent games, which best represented the 50×50 versions.

## Supporting Information

Electronic Supplementary Material S1 This supporting information extends the major findings of the paper to two different games (the extended Prisoner's Dilemma Game and the Hawk-Dove/Snowdrift game) and a wide parameter set, and gives additional methods, discussion and references.
(0.74 MB PDF)
