# Supplementary material for: Learning and Innovative Elements of Strategy Adoption Rules Expand Cooperative Network Topologies
Source: PLoS One. 2008 Apr 9;3(4):e1917. doi: 10.1371/journal.pone.0001917 (PMC2275790; doi:10.1371/journal.pone.0001917)
Supplement: Electronic Supplementary Material S1 — This supporting information extends the major findings of the paper to two different games (the extended Prisoner's Dilemma Game and the Hawk-Dove/Snowdrift game) and a wide parameter set, and gives additional methods, discussion and references. (0.74 MB PDF) [file pone.0001917.s001.pdf]

Electronic Supplementary Material (ESM) of

Learning and innovative elements of strategy adoption rules expand cooperative network topologies

Shijun Wang, Máté S. Szalay, Changshui Zhang & Peter Csermely*

*To whom correspondence should be addressed. E-mail: csermely@puskin.sote.hu

Content of this electronic supplementary material

	page
Supplementary Results	2
Supplementary Discussion	3
Supplementary References	6
Supplementary Table S1.1	9
Supplementary Table S1.2	11
Supplementary Table S1.3	13
Supplementary Figure S1.1	14
Supplementary Figure S1.2	15
Supplementary Figure S1.3	16
Supplementary Figure S1.4	17
Supplementary Figure S1.5	18
Supplementary Figure S1.6	19
Supplementary Figure S1.7	20
Supplementary Figure S1.8	21
Supplementary Figure S1.9	22
Supplementary Figure S1.10	23
Supplementary Figure S1.11	24
Supplementary Figure S1.12	25

Supplementary Text

Supplementary Results

Similarly to the case shown in Figures 1 and 2 for the best-takes-over strategy adoption rule in canonical Prisoner's Dilemma games, the three short-term strategy adoption rules (pair-wise comparison dynamics, proportional updating and best-takes-over) resulted in a rather remarkable variation of cooperator levels in Hawk-Dove games when using large number of small-world and scale-free model networks (Figures S1.1 and S1.2). For the description of game types, strategy adoption rules and model networks see Methods and refs. [1-11].

At Figure S1.1 the m=1 scale-free networks display an irregular 'phase-transition'-like phenomenon, which is most pronounced at the proportional updating strategy adoption rule but leads to a faster decay of cooperation at all short-term strategy adoption rules tested. At the construction of these m=1 scale-free networks the novel nodes are linked to the existing network with a single link only, which results in a tree-like final topology. Due to the especially large wiring-irregularity of these networks (as compared to the similarly scale-free, but more 'cross-linked' networks, where the new nodes are joined with more than one links to the existing network) a gradual change in the payoff values makes a more rapid disappearance of cooperation. At panel E of Figure S1.1 a non-monotonic behavior of p=0 networks is observed. This is derived from the extreme sensitivity of these p=0 regular networks on initial conditions, strategy update rules, etc (see references listed in Table S1.1).

Both Q-learning and the long-term versions of all three strategy adoption rules above outperformed the short-term variants resulting in a higher proportion of cooperators in Hawk-Dove games on small-world and scale-free model networks especially at high cooperation costs (Figures S1.2*A*, S1.2*B* and S1.3). Long-term strategy adoption rules (including Q-learning) were also more efficient inducers of cooperation even at high costs in modular networks (Figure S1.7). Moreover, long-term strategy adaption rules maintained cooperation even in randomly mixed populations as well as in repeatedly re-randomized networks (Figure S1.5). Interestingly, long-term strategy adoption rules (especially the long-term version of the best-takes-over strategy adoption rule) resulted in an extended range of all-cooperator outcomes in Hawk-Dove games (Figures S1.3–S1.5 and S1.7). Finally, long-term strategy adoption rules helped cooperation in canonical and extended Prisoner's Dilemma games in case of all three strategy adoption rules tried (Figure S1.6).

While short- and long-term strategy adoption rules resulted in a remarkable variation of the cooperation level in a large variety of random, regular, small-world, scale-free and modular networks in Hawk-Dove and both canonical and extended Prisoners' Dilemma games (Figures S1.1–S1.6), Q-learning induced a surprising stability of cooperation levels in all the above circumstances (Figures S1.2–S1.6). Interestingly, but expectedly, Q-learning also stabilized final cooperation levels, when games were started from a different ratio of cooperators (ranging from 10% to 90%) than the usual 50% (data not shown). When we introduced innovativity to long-term strategy adoption rules in Hawk-Dove games (for the description of these innovative strategy adoption rules see Methods) similarly to that shown for the canonical Prisoner's Dilemma game on Figure 2, cooperation levels were closer to each other in small-world and scale-free networks than their similarity observed when using only long-term, but not innovative strategy adoption rules (Figure S1.7). Importantly, innovativity alone, when applied to the best-takes-over short-term strategy adoption rule could also stabilize cooperation levels in small-world and scale-free networks (Figure S1.7*C*). When we compared different levels of innovation by changing the value of $P_{innovation}$ in our simulations (Figure S1.8), an intermediary level of innovation was proved to be optimal for the stabilization of cooperation in small-world and scale-free networks. Scale-free networks and Prisoner's Dilemma game were more sensitive to higher innovation levels than small-

world networks or Hawk-Dove games, respectively (Figure S1.8). Summarizing our results, Figures S1.9 and S1.10 show that similarly to canonical Prisoner's Dilemma games (Figure 3), both in Hawk-Dove games (Figure S1.9) and extended Prisoner's Dilemma games (Figure S1.10) long-term strategy adoption rules and innovation (including Q-learning) resulted in a stable non-zero cooperation in a large variety of network topologies in combination only.

Figure S1.11 shows the distribution of hawks (blue dots) and doves (orange dots) at the last round of a repeated Q-learning game on small-world (Figure S1.11*A* and S1.11*B*) or scale-free networks (Figure S1.11*C* and S1.11*D*) at low (Figure S1.11*A* and S1.11*C*) and high (Figure S1.11*B* and S1.11*D*) relative gain/cost (G) values. Under these conditions both hawks and doves remained isolated (see arrows). On the contrary, when Hawk-Dove games were played with any of the three short-term, non-innovative strategy adoption rules doves, but even hawks showed a tendency to form networks (Figure S1.12 and data not shown). This effect was especially pronounced for doves in both small-world and scale-free networks, as well as for hawks in small-world networks, and present, but not always that strong for hawks in scale-free networks, where hawks remained more isolated in all configurations. Interestingly, the proportional updating strategy adoption rule quite often showed an extreme behavior, when in the last round of the play all agents were either doves or hawks. This behavior was less pronounced with a larger number (2,500) of players. All the above findings were similarly observed in extended Prisoner's Dilemma games (data not shown).

Supplementary Discussion

Explaining cooperation has been a perennial challenge in a large section of scientific disciplines. The major finding of our work is that learning and innovation extend network topologies enabling cooperative behavior in the Hawk-Dove (Figures S1.1–S1.5 and S1.7–S1.9, S1.11, S1.12) and even in the more stringent Prisoner's Dilemma games (Figures 1–3, S1.6, S1.8 and S1.10). The meaning of 'learning' is extended here from the restricted sense of imitation or learning from a teacher. Learning is used in this paper to denote all types of information collection and processing to influence game strategy and behavior. Therefore, learning here includes communication, negotiation, memory and various reputation building mechanisms. Learning makes life easier, since instead of the cognitive burden to foresee and predict the 'shadow of the future' [4–6] learning allows to count on the 'shadow of the past', the experiences and information obtained on ourselves and/or other agents [12]. Likewise to our understanding of learning, the meaning of 'innovation' is extended here from the restricted sense of innovation by conscious, intelligent agents. Innovation is used in this paper to denote all irregularities in the strategy adoption process of the game. Therefore, innovation here includes errors, mutations, mistakes, noise, randomness and increased temperature besides conscious changes in game strategy adoption rules.

In the Supplementary Discussion, first we summarize the effects of network topology on cooperative behavior, then discuss the previous knowledge on the help of cooperation by learning and innovation, and, finally, we compare our findings with existing data in the literature and show their novelty and implications.

Effect of network topology on cooperation. Cooperation is not an evolutionary stable strategy [13], since in the well-mixed case, and even in simple spatial arrangements it is outcompeted by defectors. As it is clear from the data summarized in Table S1.1, the emergence of cooperation requires an extensive spatial segregation of players helping cooperative communities to develop, survive and propagate. Cooperation in repeated multi-agent games is very senitive to network topology. Cooperation becomes hindered, if the network gets over-connected [14–16]. On the contrary, high clustering [17,18], the development of fully connected cliques (especially overlapping triangles) and rather isolated communities [14,18] usually help cooperation. Heterogeneity of small-worlds and, especially,

networks with scale-free degree distribution can establish cooperation even in cases, when the costs of cooperation become exceedingly high.

However, in most spatial arrangements cooperation is rather sensitive to the strategy adoption rules of the agents, and especially to the strategy adoption rules of those agents, which are hubs, or by any other means have an influential position in the network. Moreover, minor changes in the average degree, actual degree, shortests paths, clustering coefficients or assortativity of network topology may induce a profound change in the cooperation level. Since real world networks may have rather abrupt changes in their topologies [17,20–26], it is highly important to maintain cooperation during network evolution.

Effect of learning on cooperation. From the data of Table S1.2 it is clear that learning generally helps cooperation. Cooperation can already be helped by a repeated play, assuming 'learning' even among spatially disorganized players. Memory-less or low memory strategy adoption rules do not promote cooperation efficiently. In contrast, high-memory and complex negotiation and reputation-building mechanisms (requiring the learning, conceptualization and memory of a whole database of past behaviors, rules and motives) can enormously enhance cooperation making it almost inevitable. As a summary, in the competitive world of games, it pays to learn to achieve cooperation. However, it is not helpful to know too much: if the ranges of learning and the actual games differ too much, cooperation becomes impossible [18].

Learning requires a well-developed memory and complex signaling mechanisms, which are costly. This helps the selection process in evolution [13], since 'high-quality' individuals can afford the luxury of both the extensive memory and costly signaling [27]. However, cooperation is rather widespread among bacteria, where even the 'top-quality individuals' do not have the extensive memory mentioned above. Here 'learning' is achieved by the fast succession of multiple generations. The Baldwin-effect describing the genetic (or epigenetic) fixation of those behavioral traits, which were benefitial for the individuals, may significantly promote the development of bacterial cooperation and the establishment of biofilms [28–32]. Genetically 'imprinted' aids of cooperation are also typical in higher organisms including humans. The emotional reward of cooperation uncovered by a special activation of the amygdalia region of our brains [33] may be one of the genetically stabilized mechanisms, which help the extraordinary level of human cooperation besides the complex cognitive functions, language and other determinants of human behavior.

Effect of randomness ('innovation') on cooperation. From the data of Table S1.3 it is clear that a moderate amount of randomness, 'innovation' generally helps cooperation. Many of the above learning mechanisms imply sudden changes, innovations. Bacteria need a whole set of mutations for interspecies communication (such as quorum sensing), which adapt individual organisms to the needs of cooperation in biofilms or symbiotic associations. The improved innovation in the behavior of primates and humans during games has been well documented [34–36].

An appropriate level of innovation rescues the spatial assembly of players from deadlocks, and accelerates the development of cooperation [18]. Many times noise acts in a stochastic resonance-like fashion, enabling cooperation even in cases, when cooperation could not develop in a zero-noise situation [37,38]. As a special example, the development of cooperation between members of a spatial array of oscillators (called synchrony) is grossly aided by noise [39]. Egalitarian motives also introduce innovative elements to strategy selection helping the development of cooperation [40].

However, innovation serves the development of cooperation best, if it remains a luxurious, rare event of development. Continuous 'innovations' make the system so noisy, that it looses

all the benefits of learning and spatial organization and reaches the mean-field limit of randomly selected agents with random strategy adoption rules (Table S1.3).

Comparison and novelty of our findings. In Hawk-Dove games on modified Watts-Strogatz-type small-world [2,9] and Barabasi-Albert-type [10] scale-free model networks we obtained very similar results of cooperation levels in all synchronously updated pair-wise comparison dynamics, proportional updating and best-takes-over strategy adoption rules to those of Tomassini et al. [2,3]. The success of our various 'long-term' strategy adoption rules to promote cooperation is in agreement with the success of pair-wise comparison dynamics and best-takes-over strategy adoption rules with accumulated payoffs on scale-free networks [1,3].

On the contrary to Hawk-Dove games, in the Prisoner's Dilemma game defection always has a fitness advantage over cooperation, which makes the achievement of substantial cooperation levels even more difficult. In the extended Prisoner's Dilemma games on scale-free networks [10] we obtained very similar results of cooperation levels using synchronously updated pair-wise comparison dynamics and best-takes-over strategy adoption rules to those of Tomassini et al. [3]. Similarly to the Hawk-Dove game with the extended Prisoner's Dilemma game our results with various 'long-term' strategy adoption rules on scale-free networks are in agreement with those of pair-wise comparison dynamics and best-takes-over strategy adoption rules using accumulated payoffs [1,3].

We have to note that the definition of pair-wise comparison dynamics strategy adoption rule was slightly different here, than in previous papers, and on the contrary to the non-averaged payoffs used previously, we used average payoffs [1–3], which allows only a rough comparison of these results to those obtained before, and resulted in a lower level of cooperation than that of e.g. ref. [1]. The reason we used average payoff was that this made the final level of cooperators more stable at scale-free networks even after the first 5,000 rounds of the play (data not shown). When we used non-averaged payoffs in the extended Prisoner's Dilemma game with 100,000 rounds of play, we re-gained the cooperation levels of ref. [1] at scale-free networks (m=4, data not shown). The additional papers on the subject used differently designed small-world networks or different strategy adoption rules, and therefore can not be directly compared with the current data. It is worth to mention that none of the previous papers describing multi-agent games on various networks [1–3] used the canonical Prisoner's Dilemma game, which was used obtaining our data in the main text, and which gives the most stringent condition for the development of cooperation.

As a summary, our work significantly extended earlier findings, and showed that the introduction of learning and innovation to game strategy adoption rules helps the development of cooperation of agents situated in a large variety of network topologies. Moreover, we showed that learning and innovation help cooperation separately, but act synergistically, if introduced together especially in the complex form of the reinforcement learning, Q-learning.

Interactions of learning and innovations, conclusions. Real complexity and excitement of games needs both learning and innovation. In Daytona-type car races skilled drivers use a number of reputation-building and negotiation mechanisms, and by continuously bringing novel innovations to their strategies, skilfully navigate between at least four types of games [41].

Noise is usually regarded to disturb the development of cooperation. Importantly, complex learning strategies can actually utilize noise to drive them to a higher level of cooperation. Noise may act as in the well-known cases of stochastic resonance, or stochastic focusing (with extrinsic and intrinsic noise, respectively) enabling cooperation even in cases, when it could not develop without noise. In a similar fashion, mistakes increase the efficacy of

learning [37,38,42]. Additional noise greatly helps the optimization in the simulated annealing process [43–45].

Noise not only can extend the range of cooperation to regions, where the current level of learning would not be sufficient to achieve it, but extra learning can also ‘buffer’ an increased level of noise [19]. Thus, learning and innovation act side-by-side and – in gross terms – correct the deficiencies of the other. Learning and innovation also cooperate in the Baldwin effect, where beneficial innovations (in the form of mutations) are selected by the inter-generational ‘meta-learning’ process of evolution [28–32]. Mutual learning not only makes innovation tolerable, but also provokes a higher level of innovation to surpass the other agent [36].

Our work added the important point to this emerging picture that the cooperation between learning and innovation to achieve cooperation also works in the extension and buffering of those network configurations, where cooperation becomes possible.

Supplementary References

1. Santos FC, Pacheco JM (2005) Phys. Rev. Lett. 95: 098104.
2. Tomassini M, Luthi L, Giacobini M (2006) Phys. Rev. E 73: 016132.
3. Tomassini M, Pestelacci E, Luthi L (2007) Int. J. Mod. Physics C 18: 1173–1186.
4. Axelrod R, Hamilton WD (1981) Science 211: 1390–1396.
5. Axelrod R (1984) The evolution of cooperation. (Basic Books, New York).
6. Cohen MD, Riolo RL, Axelrod R (2001) Rationality Society 13: 5–32.
7. Watkins CJCH, Dayan P (1992) Machine Learning 8: 279–292.
8. Sutton RS, Barto AG (1999) Reinforcement learning: an introduction. (Cambridge University Press, Cambridge).
9. Watts DJ, Strogatz SH (1998) Nature 393: 440–442.
10. Barabasi AL, Albert R (1999) Science 286: 509–512.
11. Girvan M, Newman MEJ (2002) Proc. Natl. Acad. Sci. USA 99: 7821–7826.
12. Macy MW, Flache A (2002) Proc. Natl. Acad. Sci. USA 99: 7229–7236.
13. Maynard-Smith J (1982) Evolution and the theory of games. (Cambridge University Press, Cambridge).
14. Ohtsuki H, Hauert C, Lieberman E, Nowak MA (2006) Nature 441: 502–505.
15. Tang CL, Wang WX, Wu X, Wang BH (2006) Eur. J. Phys. B 53: 411–415.
16. Chen XJ, Fu F, Wang L (2007) Physica A 378: 512–518.
17. Santos FC, Rodrigues JF, Pacheco JM (2006) Proc. R. Soc. B 273: 51–55.
18. Ifti M, Killingback T, Doebeli M (2004) J. Theor. Biol. 231: 97–106.
19. Hruschka DJ, Henrich J (2006) J. Theor. Biol. 239: 1–15.
20. Derenyi I, Farkas I, Palla G, Vicsek T (2004) Physica A 334: 583–590.
21. Biely C, Dragosits K, Thurner S (2007) Physica D 228: 40–48.
22. Eguíluz VM, Zimmermann MG, Cela-Conde CJ, Miguel MS (2005) Am. J. Sociol. 110: 977–1008.
23. Zimmermann MG, Eguíluz VM (2005) Phys. Rev. E 72: 056118.
24. Zimmermann MG, Eguíluz VM, Miguel MS (2004) Phys. Rev. E 69: 065102.
25. Santos FC, Pacheco JM, Lenaerts T (2006) Proc. Natl Acad. Sci. USA 103: 3490–3494.
26. Santos FC, Pacheco JM, Lenaerts T (2006) PLOS Comput. Biol. 2: e140.
27. Fehr E, Fishbacher U (2003) Nature 425: 785–791.
28. Baldwin JM (1896) Am. Nat. 30: 441–451.
29. Baldwin JM (1896) Am. Nat. 30: 536–553.
30. Simpson GG (1953) Evolution 7: 110–117.
31. Ancel LW (1999) J. Theor. Biol. 196: 197–209.
32. Suzuki R, Arita T (2004) BioSystems 77: 57–71.
33. Rilling JK, Glenn AL, Jairam MR, Pagnoni G, Goldsmith DR, Elfenbein HA, Lilienfeld SO (2007) Biol. Psych. 61: 1260–1271.
34. Lee D, Conroy ML, McGreevy BP, Barraclough DJ (2004) Cogn. Brain Res. 22: 45–58.
35. Rapoport A, Budescu D (1992) J. Exp. Psychol. 121: 352–363.
36. Soltani A, Lee D, Wang XJ (2006) Neural Networks 19: 1075–1090.

37. Traulsen A, Röhl T, Schuster HG (2004) Phys. Rev. Lett. 93: 028701.
38. Eriksson A, Lindgren K (2005) J. Theor. Biol. 232: 399–409.
39. Braiman Y, Lindner JF, Ditto WL (1995) Nature 378: 465–467.
40. Dawes CT, Fowler JH, Johnson T, McElreath R, Smirnov O (2007) Nature 446: 794–796.
41. Ronfeldt D (2000) First Monday 5: 2.
42. Nowak MA, Plotkin JB, Krakauer DC (1999) J. Theor. Biol. 200: 147–162.
43. Barakat MT, Dean PM (1990) J. Comput. Aided Mol. Des. 4: 317–330.
44. Hart TN, Read RJ (1992) Proteins 13: 206–222.
45. Hellinga HW, Richards FM (1994) Proc. Natl. Acad. Sci. USA 91: 5803–5807.
46. Nowak MA, May RM (1992) Nature 359: 826–829.
47. Hauert C, Doebeli M (2004) Nature 428: 643–646.
48. Sysi-Aho M, Saramaki J, Kertész J, Kaski K (2005) Eur. Phys. J. B 44: 129–135.
49. Vainstein MH, Arenzon JJ (2001) Phys. Rev. E 64: 051905.
50. Vukov J, Szabó G (2005) Phys. Rev. E 71: 036133.
51. Szöllősi GJ, Derényi I (2007) www.arxiv.org/0704.0357.
52. Vukov J, Szabó G, Szolnoki A (2006) Phys. Rev. E 73: 067103.
53. Ebel H, Bornholdt S (2002) Phys. Rev. E 66: 056118.
54. Santos FC, Rodrigues JF, Pacheco JM (2005) Phys. Rev. E 72: 056128.
55. Ren J, Wang W, Qi F (2007) Phys. Rev. E 75: 045101.
56. Kim BJ, Trusina A, Holme P, Minnhagen P, Chung JS, Choi MY (2002) Phys. Rev. E 66: 021907.
57. Chen XJ, Fu F, Wang L (2007) www.arxiv.org/physics/0701318.
58. Gómez-Gardenes J, Campillo M, Floria LM, Moreno Y (2007) Phys. Rev. Lett. 98: 108103.
59. Wu ZX, Guan JY, Xu XJ, Wang YH (2007) Physica A 379: 672–680.
60. Holme P, Trusina A, Kim BJ, Minnhagen P (2003) Phys. Rev. E 68: 030901.
61. Brauchli C, Killingback T, Doebeli M (1999) J. Theor. Biol. 200: 405–417.
62. Lindgren K, Nordahl MG (1994) Physica D 75: 292–319.
63. Nowak MA, Sigmund K (1992) Nature 355: 250–253.
64. Kraines D, Kraines V (1989) Theory Decision 26: 47–79.
65. Nowak MA, Sigmund K (1993) Nature 364: 56–58.
66. Nowak MA, Sigmund K (1993) Proc. Natl. Acad. Sci. USA 90: 5091–5094.
67. Crowley PH, Provencher L, Sloane S, Dugatkin LA, Spohn B, Rogers L, Alfieri M (1996) BioSystems 37: 49–66.
68. Neill DB (2001) J. Theor. Biol. 211: 159–180.
69. Wang WX, Ren J, Chen G, Wang BH (2006) Phys. Rev. E 74: 056113.
70. Ochoa JD (2007) Physica A 386: 752–755.
71. Qin SM, Chen Y, Zhao XY, Shi J (2008) arXiv:0801.2827.
72. Helbing D, Schönhof M, Stark HU, Holyst JA (2005) Adv. Complex Syst. 8: 87–116.
73. Tan M (1993) Proc. 10th Intl. Conf. Machine Learning 330–337.
74. Gutnisky DA, Zanutto BS (2004) Artif. Life 10: 65–81.
75. Riedmiller M, Moore A, Schneider J (2000) in Balancing reactivity and Social Deliberation in Multiagent Systems, eds Hannebauer M, Wendler J, Pagello E (Springer-Verlag, Heidelberg), pp 137–149.
76. Zamora J, Millán JR, Murciano A (1998) Biol. Cybern. 78: 197–205.
77. Skyrms B, Pemantle R (2000) Proc. Natl. Acad. Sci. USA 97: 9340–9346.
78. Downs A, Smith T (2004) J. Autism Dev. Disord. 34: 625–635.
79. Milinski M (1987) Nature 325: 433–435.
80. Milinski M, Wedekind C (1998) Proc. Natl. Acad. Sci. USA 95: 13755–13758.
81. Packer C, Pusey AE (1997) Sci. Am. 276: 32–39.
82. Sanabria F, Baker F, Rachlin H (2003) Learning Behav. 31: 318–331.
83. Seyfarth RM, Cheney DL (1984) Nature 308: 541–543.
84. Stephens DW, McLinn CM, Stevens JR (2002) Science 298: 2216–2218.
85. Wilkinson GS (1984) Nature 308: 181–184.
86. Wilkinson GS (1986) Anim. Behav. 34: 1880–1889.
87. Brinck I, Gardenfors P (2003) Mind Language 18: 484–501.
88. Brown SP, Johnstone RA (2001) Prog. Biol. Sci. 268: 961–965.
89. Huberman BA, Lukose RM (1997) Science 277: 535–537.
90. Kiesler S, Sproull L, Waters K (1996) J. Pers. Soc. Psychol. 70: 47–65.
91. McNamara JM, Gasson CE, Houston AI (1999) Nature 401: 368–341.
92. Miller JH, Butts CT, Rode D (2002) J. Econ. Behav. Org. 47: 179–195.
93. Turner PE, Chao L (1999) Nature 398: 441–443.

94. Uzzi B (1997) Adm. Sci. Quart. 42: 35–67.
95. Benjamin SC, Hayden PM (2001) Phys. Rev. A 64: 030301.
96. Bolton GE, Katok E, Ockenfels A (2004) Management Sci. 50: 1587–1602.
97. Riolo RL, Cohen MD, Axelrod R (2001) Nature 414: 441–443.
98. Sigmund K, Hauert C, Nowak MA (2001) Proc. Natl. Acad. Sci. USA 98: 10757–10762.
99. Sella G (1988) Biol. Bull. 175: 212–217.
100. Silverstein A, Cross D, Brown J, Rachlin H (1998) J. Behav. Decision Making 11: 123–138.
101. Wong RY, Hong YY (2005) Psychol. Sci. 16: 429–434.
102. Nowak MA, Bonhoeffer S, May RM (1994) Int. J. Bifurcat. Chaos 4: 33–56.
103. Mukherji A, Rajan V, Slagle JR (1996) Nature 379: 125–126.
104. Nowak MA, Bonhoffer S, May RM (1996) Nature 379: 126.
105. Perc M (2006) Europhys. Lett. 75: 841–846.
106. Masuda N, Aihara K (2003) Phys. Lett. A 313: 55–61.
107. Szabó G, Hauert C (2002) Phys. Rev. Lett. 89: 118101.
108. Grim P (1996) BioSystems 37: 3–17.
109. Hauert C, De Monte S, Hofbauer J, Sigmund K (2002) Science 296: 1129–1132.
110. Szabó G, Hauert C (2002) Phys. Rev. E 66: 062903.
111. Szabó G, Vukov J (2004) Phys. Rev. E 69: 036107.
112. Hauert C, Traulsen A, Brandt H, Nowak MA, Sigmund K (2007) Science 316: 1905–1907.
113. McNamara JM, Barta Z, Fromhage L, Houston AI (2008) Nature 451: 189–192.
114. Rockenbach B, Milinski M (2006) Nature 444: 718–723.
115. Sandholm TW, Crites RH (1996) BioSystems 37: 147–166.
116. Batagelj V, Mrvar A (1998) Connections 21: 47–57.

Supplementary Tables

Table S1.1. Effect of network topology on cooperation

**Network topology**	**Effect on cooperation**	**Games; strategy adoption rules**	**Agents** (players)	**References**
**Lattice**	**Sensitive to strategy adoption rules and topology** (cooperation level is very sensitive on strategy adoption rules, high degree ***inhibits*** cooperation)	HD, PD[a]	Simulation	14, 46–48
**Lattice with dilution** (with empty spaces)	**Helps** (localized groups of cooperators emerge better)	PD	Simulation	49
**Lattice with hierarchical layers**	**Helps** (at top level, if the number of levels is lower than 4; in middle layers otherwise)	PD	Simulation	50, 51
**Regular random graphs**	**Sensitive to topology** (triangles help, loops>3 and high degree ***inhibit*** cooperation)	PD	Simulation	14, 52
**Random graphs**	**Sensitive to topology, long-lasting avalanches may develop** (high degree ***inhibits*** cooperation)	PD	Simulation	14, 15, 53
**Small-world** (Watts-Strogatz-type)	**Mostly helps** (helps the spread of cooperation + introduces heterogeneity to stabilize it, high degree ***inhibits*** cooperation)	PD	Simulation	14, 15, 54, 55
**Small-world** (randomly replaced edges)	**Sensitive to strategy adoption rules** (very sensitive to the applied strategy adoption rules)	HD	Simulation	2, 3
**Small-world** (Watts-Strogatz-type) **with an influential node**	**Destabilizes** (the central node is very sensitive for attacks by defectors)	PD	Simulation	56
**Homogenous small-world** (degree is kept identical)	**Sensitive to topology and temptation level** (at small temptation helps the attack of defectors via shortcuts, helps at high temptation)	PD	Simulation	54, 55
**Scale-free** (Barabasi-Albert-type)	**Sensitive to to strategy adoption rules** (hubs stabilize cooperation but make it vulnerable to targeted attacks, clusters and loops help, sensitive to strategy adoption rules, high degree ***inhibits*** cooperation)	HD, PD; pair-wise comparison dynamics, imitation of the best	Simulation	1, 3, 14–16, 57–59

Table S1.1. Effect of network topology on cooperation (continued)

**Network topology**	**Effect on cooperation**	**Games; strategy adoption rules**	**Agents** (players)	**References**
**Scale-free with hierarchy** (Ravasz-Barabasi-type hierarchy)	**Inhibits** (makes it very sensitive for the attack of defectors)	PD	Simulation	50
**Scale-free with communities**	**Helps** (isolated communities help intra-community cooperation)	PD	Simulation	16
**Real world networks**	**Generally helps** (small-worlds and hierarchy help cooperation)	PD	Internet communities, emails, karate club	60
**Dynamic** (evolves during the game)	**Generally helps** (a small-world and hierarchy develops, which stabilizes cooperation, a slower reaction to new information is beneficial)	PD	Simulation	17, 21–26

[a]HD = Hawk-Dove (Snowdrift, Chicken) game; PD = Prisoner's Dilemma game (please note that in this supplementary table we did not discriminate between conventional and cellular automata-type games, where in the latter simulating evolution agents 'die', and are occasionally replaced; in our simulations we used only 'conventional' games, where agent-replacement was not allowed).

Table S1.2. Effect of learning on cooperation

**Type of learning**[a]	**Effect on cooperation**	**Networks; games; strategy adoption rules**	**Agents** (players)	**References**
**One-step learning strategy adoption rules**	**Help** (increases cooperation in repeated multi-agent games)	Lattice; PD[b]; Tit-for-tat strategy adoption rule and its generous versions[c]	Simulation	61–63
**Two-step learning strategy adoption rules**[d]	**Help** (make cooperation rather resistant to noise → often win against Tit-for-tat)	Lattice; PD; Pavlov strategy adoption rule and its generous versions[c]	Simulation	61–66
**Extended learning strategy adoption rules** (3 or more steps)	**Help** (each additional memory unit contributes less to the increase of cooperation)	Lattice, scale-free; HD[b], PD, alternating PD with noise; higher memory 'Firm Pavlov', 'Meta-Pavlov' strategy adoption rules	Simulation	32, 62, 67–71
**Complex learning strategy adoption rules** (adaptive learning, operant conditioning, preferential learning, Q-learning, reinforcement learning)	**Help** (are not only resistant to noise but can exploit noise to drift towards cooperation, reinforcement learning based on local or global information enables sophisticated strategy adoption rules to emerge and allows efficient network formation)	Lattice, scale-free; HD, matching pennies game, PD; pair-wise comparison dynamics strategy adoption rule	Simulation, primates, humans	12, 36, 37, 46, 72–77
**Natural learning processes**	**Help** (fishes, monkeys remember their cooperators; birds learn cooperation with feedback signals or accumulated payoffs; lions learn cooperative hunting to capture fast prey; vampire bats share blood by regurgitation; students are more successful using complex Pavlov strategy adoption rules than tit-for-tat, which is the default, if their memory capacity is compromised disfavoring cooperation; subjects with psychopathy disorders have a deficit of emotional reward for cooperation, which can be corrected by learning)	PD (interfering Memory game)	Guppies, birds, vampire bats, lions, monkeys, humans (controls, subjects with psychopathy, autism, or attention-deficit hyperactivity disorder)	33, 78–86

Table S1.2. Effect of learning on cooperation (continued)

**Type of learning**[a]	**Effect on cooperation**	**Networks; games; strategy adoption rules**	**Agents** (players)	**References**
**Communication, negotiation**	**Help** (viruses lack communication and cooperation; quorum sensing is required for bacterial biofilm formation; avoidance of discussion blocks cooperation; complex communication allows better cooperation; feedback eases internet and traffic congestion; firm's market image helps cooperative response; description of future goals greatly enhances cooperation)	PD, 'game of sexes', biofilm formation, internet usage, car-race, trade	Simulation, viruses, bacteria, humans, firms	41, 72, 73, 87–94
**Quantum entanglement** ('quantum communication')	**Helps** (quantum bits, 'qubits' enable a continuous cooperation, which works as a contract)	Quantum minority game, quantum PD	Simulation	95
**Tag, reputation-building**	**Help** (establishing and learning tags and reputation help cooperators to detect each other – even without memory – and build communities)	Donation game, PD, ultimatum game, car-race, e-trade	Simulation, humans	19, 27, 41, 67, 96–98
**Evolutionary preserved recognition** (using the Baldwin-effect)	**Helps** (enables the detection and avoidance of cheaters; the learned habbit is selected and fixed by evolution)	Hermaphrodites exchanging eggs	Hermaphrodite worms	99
**Memory of cooperation patterns** (cultural context)	**Helps** (cooperation in previous games; cooperative educational or cultural traits)	Intergenerational public good game, PD, ultimatum game	Humans	12, 27, 100, 101

[a]The term 'learning' is used here in the sense of the collection and use of information influencing game strategy adoption rules and behavior, and not in the restricted sense of imitation, or directed information-flow from a dominant source (the teacher). Therefore, learning here includes communication, negotiation, memory, label-assignment and label-recognition, etc.

[b]HD = Hawk-Dove (Snowdrift, Chicken) game; PD = Prisoner's Dilemma game (please note that in this supplementary table we did not discriminate between conventional and cellular automata-type games, where in the latter simulating evolution agents 'die', and are occasionally replaced; in our simulations we used only 'conventional' games, where agent-replacement was not allowed).

[c]Tit-for-tat = this strategy adoption rule copies the opponent's step in the previous round; Pavlov = a 'win stay – lose shift' strategy adoption rule; generous strategy adoption rules = allow 'extra' cooperation options with a given probability.

[d]These strategy adoption rules are interchangeably called as 'memory-one' or 'memory-two' strategy adoption rules referring to the fact that e.g. in the Pavlov strategy adoption rule agents remember the outcome of only the last step ('memory-one') but that of both players ('memory-two').

Table S1.3. Effect of innovation on cooperation

**Type of innovation**[a]	**Effect on cooperation**	**Networks; games; strategy adoption rules**	**Agents** (players)	**References**
**Topological irregularities** (empty sites = 'sterile defectors', small-world shortcuts, hubs)	**Mostly help** (see Table 1, block the spread of defection, however high degree ***inhibits*** cooperation and irregularities make it ***sensitive*** for strategy adoption rules)	Lattice, small-world; HD, PD[b]	Simulation	49, 102 and Table S1.1
**Low noise** (random noise, errors, mistakes, the 'trembling hand')	**Helps** (at low levels resolves deadlocks, at high levels ***inhibits*** cooperation)	Evolutionary language learning game, ultimatum game	Simulation	42, 98
**High noise** (random noise, errors, mistakes, the 'trembling hand')	**Inhibits** (PD game is noise-sensitive, especially on lattices, where noise makes cooperator boundaries irregular)	Lattice; PD	Simulation	52, 102–104
**Pink noise** (chaotic changes in environment affecting payoff)	**Mostly helps** (smaller, but reliable payoffs become more attractive)	Lattice; PD	Simulation	105
**Random elements in strategy adoption rules** (strategy selection, payoff determination, etc.)	**Help** (at low levels resolve deadlocks, at high levels inhibit cooperation)	Lattice, random, small-world; HD, PD	Simulation	48, 50, 55, 106, 107
**Random extra cooperation in strategy adoption rules**	**Helps**	Lattice; PD; Generous tit-for-tat, 'double-generous-tit-for-tat'	Simulation	65, 108
**Mutation of strategy adoption rules**	**Helps** (may re-introduce cooperation)	Lattice; PD	Simulation	66
**Extra loner strategy adoption rule**[c]	**Helps** (even for large temptation values)	Lattice, small-world; PD, public good game	Simulation	107, 109–112
**Quantum probabilistic strategies**	**Help** (ancillary quantum bits, 'qubits' enable to use 'mixed' strategies)	Quantum minority game, quantum PD	Simulation	95
**Random elements in strategy adoption rules**	**Help** (increased when playing games)	matching pennies game, PD and other social dilemma games	Simulation, humans, primates	34–37, 113
**Mixed strategies**	**Help** (reputation building is supplemented with costly punishment)	PD	Humans	114
**Egalitarian motives**	**Help** (help the development of reciprocity)	Public good game	Humans	40

[a]The term 'innovation' is used here in the sense of irregularities in the process of the game. Therefore, innovation here includes errors, mutations, mistakes, noise, randomness and increased temperature besides the *senso stricto* innovation of conscious, intelligent agents.

[b]HD = Hawk-Dove (Snowdrift, Chicken) game; PD = Prisoner's Dilemma game (please note that in this supplementary table we did not discriminate between conventional and cellular automata-type games, where in the latter simulating evolution agents 'die', and are occasionally replaced; in our simulations we used only 'conventional' games, where agent-replacement was not allowed).

[c]Loners do not participate in the game and share the income with the co-player.

Supplementary Figures

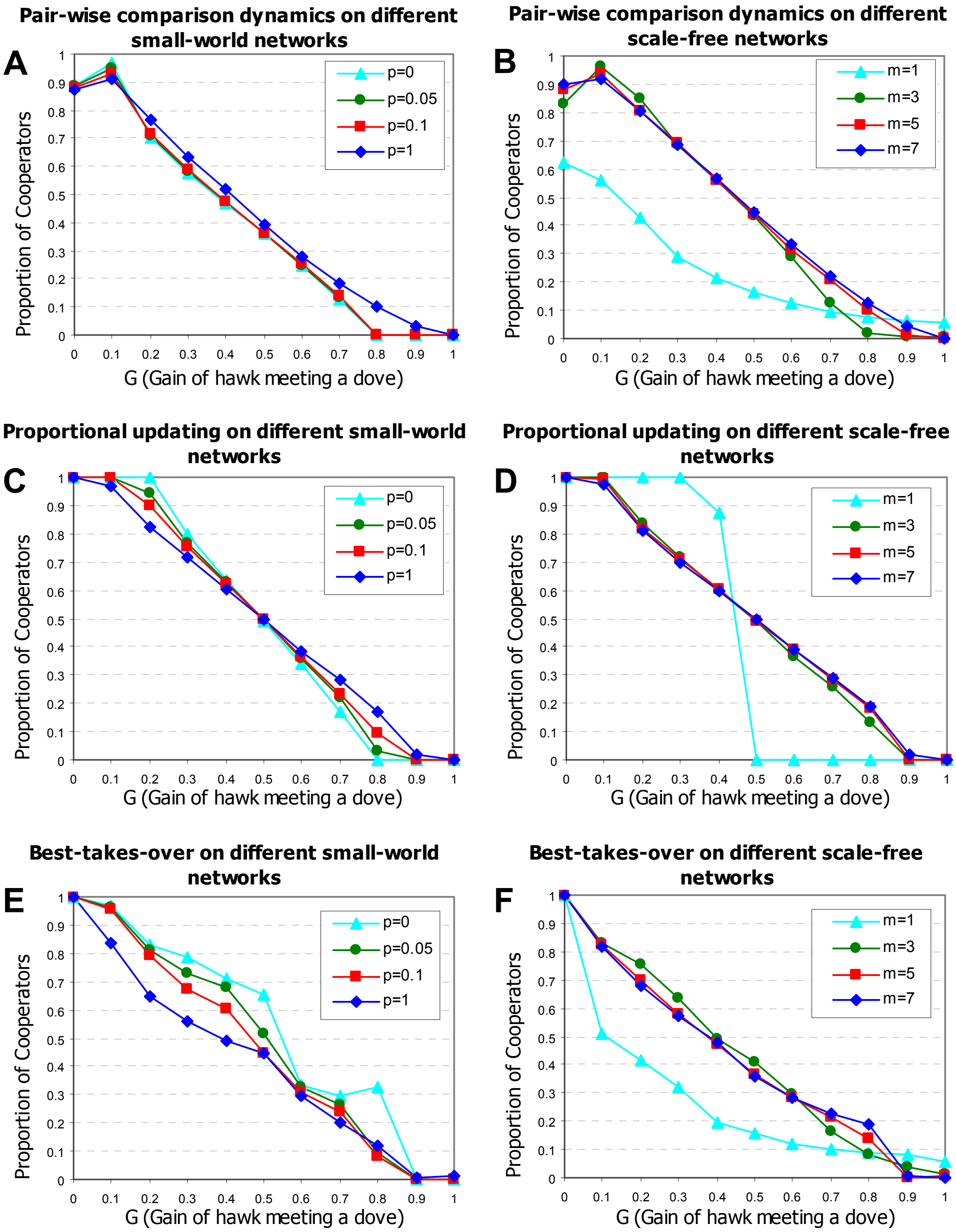

Figure S1.1. Variation of cooperation level using short-term, non-innovative strategy adoption rules in Hawk-Dove games on small-world and scale-free networks. The modified Watts-Strogatz small-world networks (Panels *A, C* and *E*) were built on a 50 x 50 lattice, where each node was connected to its eight nearest neighbors. The rewiring probability of the regular links was 0 (pale blue triangles), 0.05 (green circles), 0.1 (red squares) and 1 (dark blue diamonds). The Barabasi-Albert scale-free networks (Panels *B*, *D* and *F*) also contained 2,500 nodes, where at each construction step a new node was added with m=1 (pale blue triangles), m=3 (green circles), m=5 (red squares) or m=7 (dark blue diamonds) new links attached to the existing nodes. For the description of the networks, Hawk-Dove games and the three different strategy adoption rules, the pair-wise comparison dynamics (Panels *A* and *B*), the proportional updating (Panels *C* and *D*) and the best-takes-over strategy adoption rules (Panels *E* and *F*), see Methods. For each strategy adoption rule and *G* values (representing the gain of hawk meeting a dove, see Methods), 100 random runs of 5,000 time steps were executed.

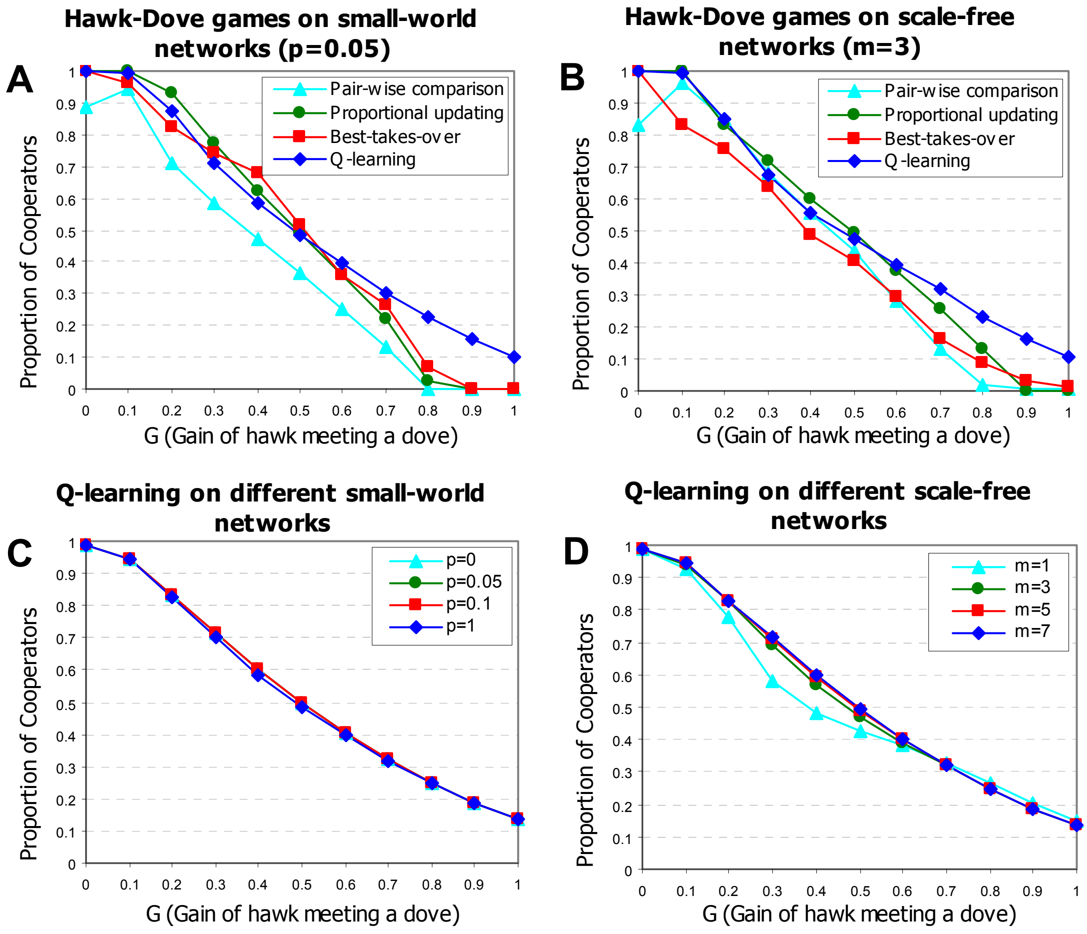

Figure S1.2. Q-learning improves and stabilizes the cooperation of agents forming small-world and scale-free networks in Hawk-Dove games. *A***,** The modified Watts-Strogatz small-world networks [2] were built on a 50 x 50 lattice, where each node was connected to its eight nearest neighbors. The rewiring probability of the regular links was 0.05. *B***,** The Barabasi-Albert scale-free networks [10] also contained 2,500 nodes, where at each construction step a new node was added with m=3 new links attached to the existing nodes. For the description of the Hawk-Dove games and the four different strategy adoption rules, pair-wise comparison dynamics (pale blue triangles), proportional updating (green circles), best-takes-over (red squares) and Q-learning (dark blue diamonds) see Methods. *C***,** The rewiring probability of the small-world network of panel *A* was 0 (regular network, pale blue triangles), 0.05 (small-world, green circles), 0.1 (small-world, red rectangles) and 1 (random network, dark blue diamonds). *D***,** The number of nodes added to the existing nodes of the scale-free network of *B* was varied between 1 and 7. For each strategy adoption rule and *G* values (representing the gain of hawk meeting a dove, see Methods), 100 random runs of 5,000 time steps were executed.

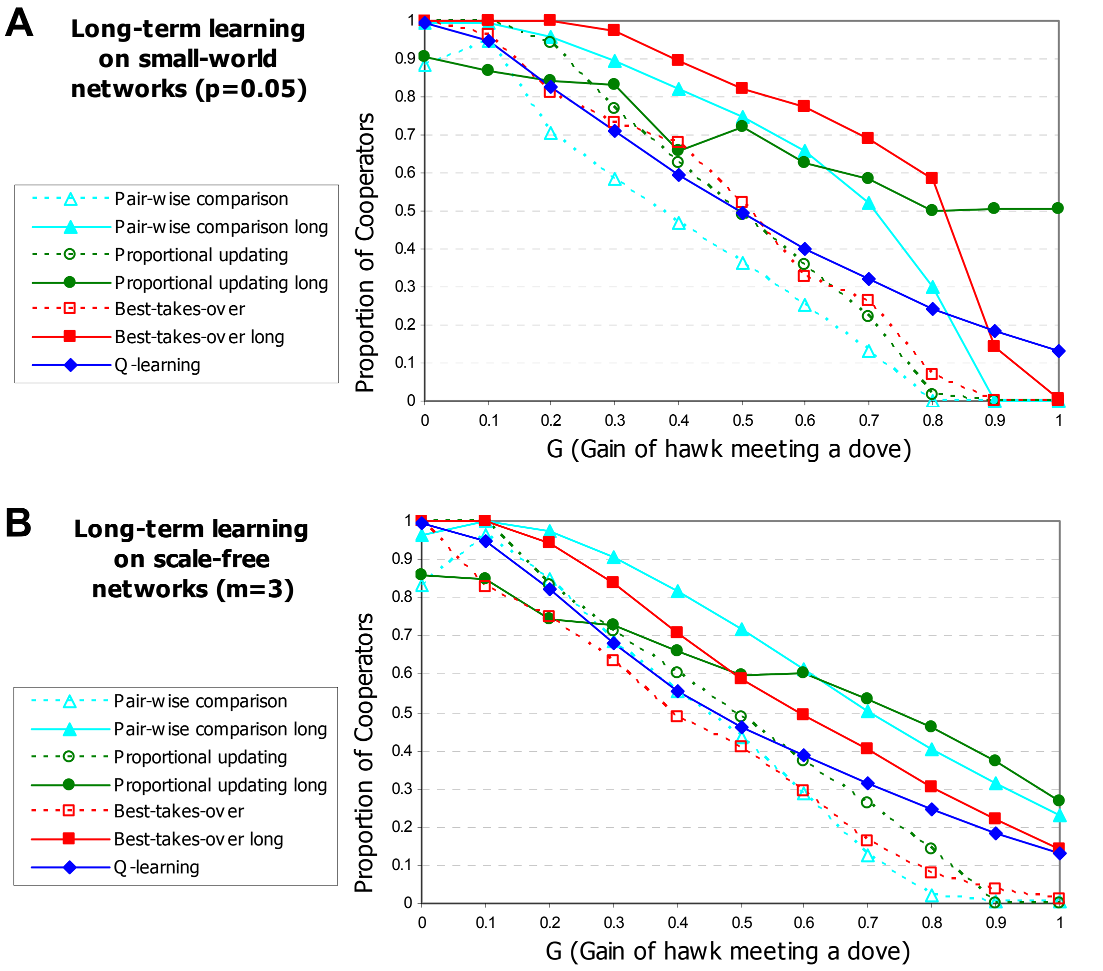

Figure S1.3. Long-term learning strategy adoption rules help cooperation in Hawk-Dove games played on various networks. For the description of the small-world [2] and scale-free [10] networks, the Hawk-Dove game and the different strategy adoption rules, pair-wise comparison dynamics (pale blue open triangles and dashed line), proportional updating (green open circles and dashed line), best-takes-over (red open squares and dashed line), Q-learning (dark blue diamonds and solid line) pair-wise comparison dynamics long (pale blue filled triangles and solid line), proportional updating long (green filled circles and solid line) and best-takes-over long (red filled squares and solid line) strategy adoption rules see Methods. *A*, Long-term learning strategy adoption rules on small-world networks with a rewiring probability of 0.05. *B*, Long-term learning strategy adoption rules on scale-free networks with m=3. For each game strategy adoption rule and *G* values (representing the gain of hawk meeting a dove, see Methods), 100 random runs of 5,000 time steps were executed.

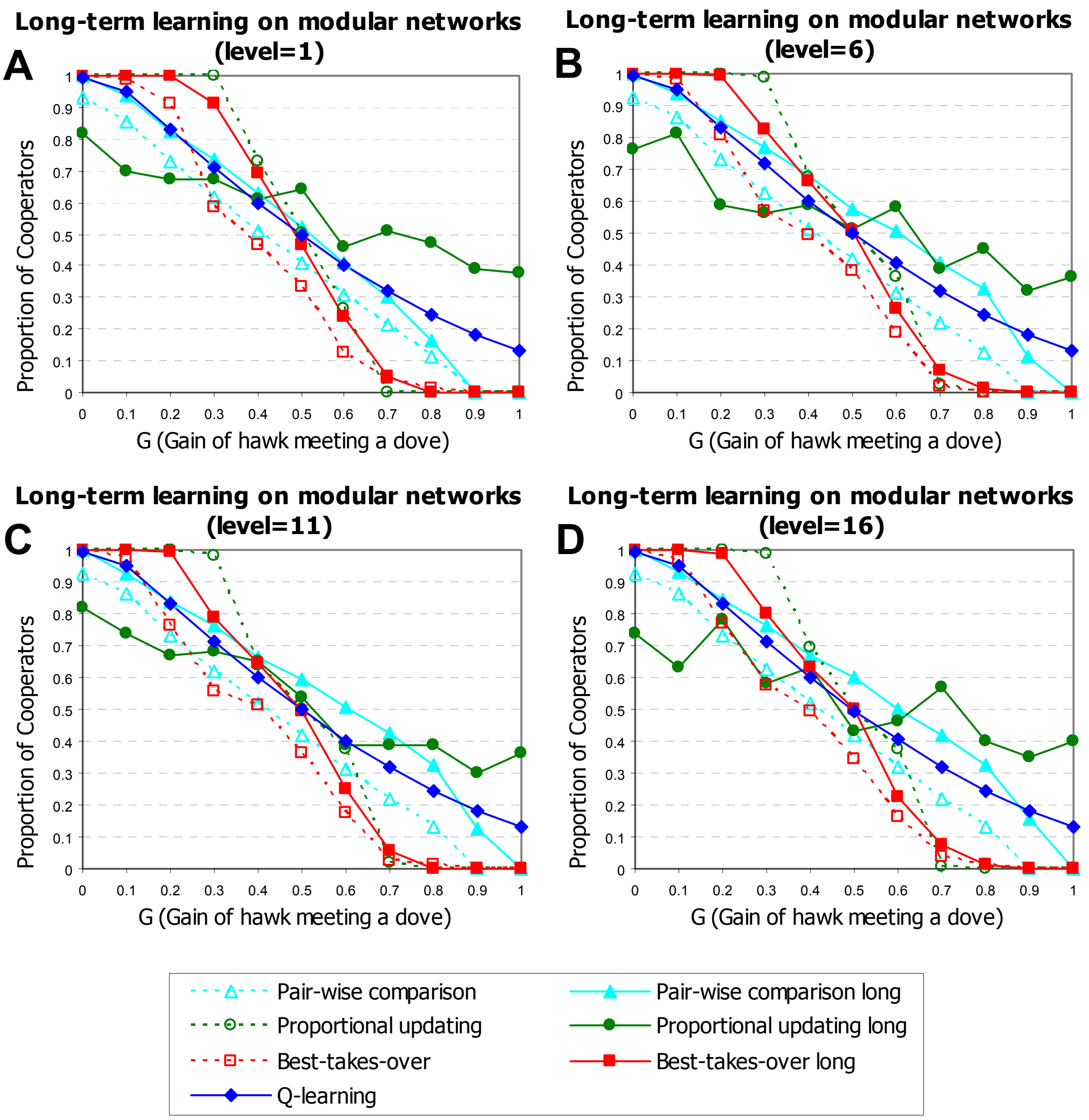

Figure S1.4. Long-term learning strategy adoption rules help cooperation in Hawk-Dove games played on modular networks. In the modular networks described by Girvan and Newman [11] each network had a scale-free degree distribution, contained 128 nodes and was divided into 4 communities. The average degree was 16. Panels *A* through *D* show the % of cooperation when playing on Girvan-Newman modular networks with levels 1, 5, 10 or 16, respectively, where 'level 1' means that for each node in the network, the expected number of links between a node and the nodes which are in other communities was 1. With increasing 'level' the community structure died down gradually. For the description of the Hawk-Dove game and the different strategy adoption rules, pair-wise comparison dynamics (pale blue open triangles and dashed line), proportional updating (green open circles and dashed line), best-takes-over (red open squares and dashed line), Q-learning (dark blue filled diamonds and solid line) pair-wise comparison dynamics long (pale blue filled triangles and solid line), proportional updating long (green filled circles and solid line) and best-takes-over long (red filled squares and solid line) strategy adoption rules see Methods. For each game strategy adoption rule and *G* values runs on 100 Girvan-Newman-type modular networks of 5,000 time steps were executed.

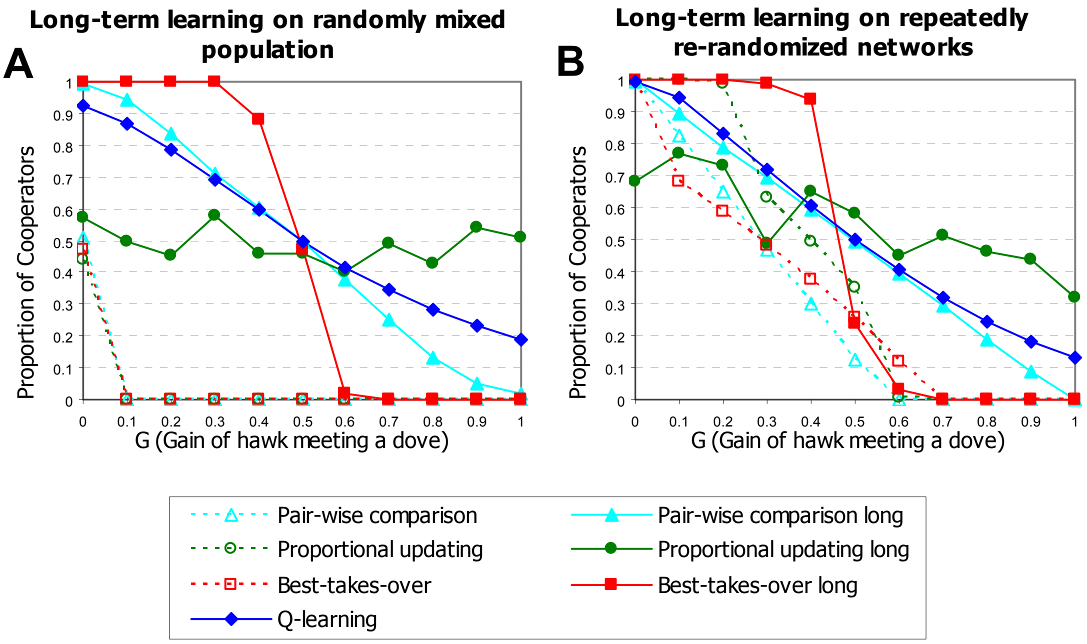

Figure S1.5. Long-term learning strategy adoption rules help cooperation in Hawk-Dove games on randomly mixed population and on repeatedly re-randomized networks. For the description of the Hawk-Dove game and the different strategy adoption rules, pair-wise comparison dynamics (pale blue open triangles and dashed line), proportional updating (green open circles and dashed line), best-takes-over (red open squares and dashed line), Q-learning (dark blue filled diamonds and solid line) pair-wise comparison dynamics long (pale blue filled triangles and solid line), proportional updating long (green filled circles and solid line) and best-takes-over long (red filled squares and solid line) strategy adoption rules see Methods. *A*, Games between two randomly selected agents from 100 total. For each game strategy adoption rule and *G* values, 100 random runs of 100,000 time steps were executed. *B*, Before each individual rounds of the repeated Hawk-Dove game, we generated a new random graph of the agents with a connection probability, p=0.02, where the number of agents was 200. In this way for a specific agent, its neighbors changed in each round of game. For each game strategy adoption rule and *G* values (representing the gain of hawk meeting a dove, see Methods), 100 random runs of 5,000 time steps were executed.

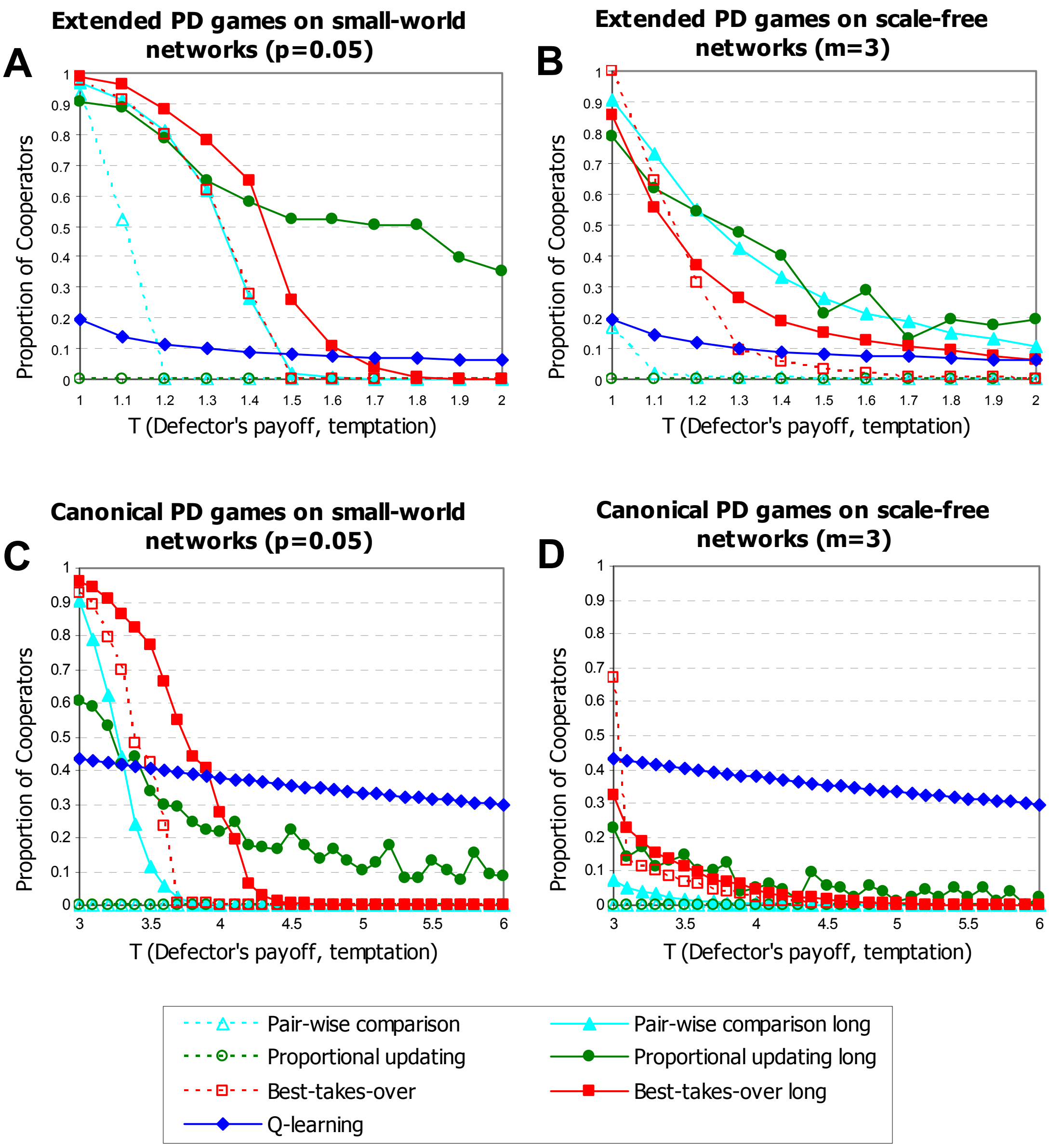

Figure S1.6. Long-term learning strategy adoption rules help cooperation in both canonical and extended Prisoner's Dilemma games played on small-world and scale-free networks. The small-world (panels *A* and *C*, [2]) and scale-free (panels *B* and *D*, [10]) networks were built as described in the Methods. For the description of the Prisoner's Dilemma games and the different strategy adoption rules, pair-wise comparison dynamics (pale blue open triangles and dashed line), proportional updating (green open circles and dashed line), best-takes-over (red open squares and dashed line), Q-learning (dark blue filled diamonds and solid line) pair-wise comparison dynamics long (pale blue filled triangles and solid line), proportional updating long (green filled circles and solid line) and best-takes-over long (red filled squares and solid line) strategy adoption rules see Methods. Panels *A* and *B*, extended Prisoner's Dilemma games ($R=1, P=0, S=0$ T was changed from 1 to 2; 1). Panels *C* and *D*, canonical Prisoner's Dilemma games ($R=3, P=1, S=0$ T was changed from 3 to 6; [6]). In the canonical Prisoner's Dilemma games when using the Q-learning, the initial annealing temperature was set to 10,000 to extend the annealing process [115]). For each game strategy adoption rule and T values 100 random runs of 5,000 time steps were executed.

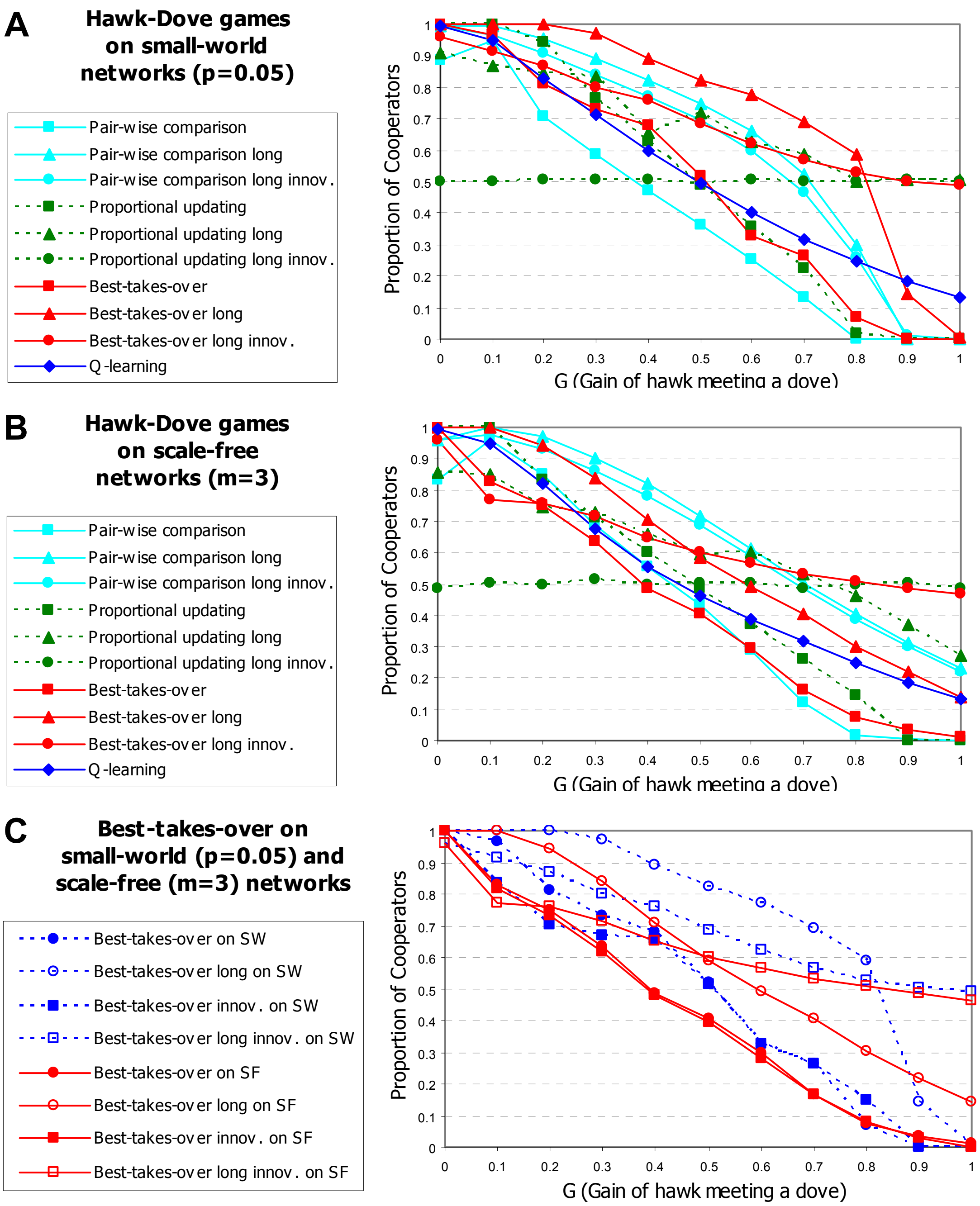

Figure S1.7. Comparison of innovative strategy adoption rules in Hawk-Dove games on small-world and scale-free networks. The small-world (*A* and *C* blue symbols and dashed lines) and scale-free (*B* and *C* red symbols and solid lines) networks were built as described in Methods. For the description of the Hawk-Dove game and the different strategy adoption rules, pair-wise comparison dynamics (pale blue filled squares, solid line), pair-wise comparison dynamics long (pale blue filled triangles, solid line), pair-wise comparison dynamics long innovative (pale blue filled circles, solid line), proportional updating (green filled squares, dashed line), proportional updating long (green filled triangles, dashed line), proportional updating long innovative (green filled circles, dashed line), best-takes-over (on panel *A* and *B*: red filled squares, on panel *C*: filled circles), best-takes-over long (on panel *A* and *B*: red filled triangles, on panel *C*: open circles), best-takes-over innovative (on panel *C*: filled squares), best-takes-over long innovative (on panel *A* and *B*: red filled circles, on panel *C*: open squares), and Q-learning (blue filled diamonds) strategy adoption rules, see Methods. For each game strategy adoption rule and *G* values (representing the gain of hawk meeting a dove, see Methods), 100 random runs of 5,000 time steps were executed.

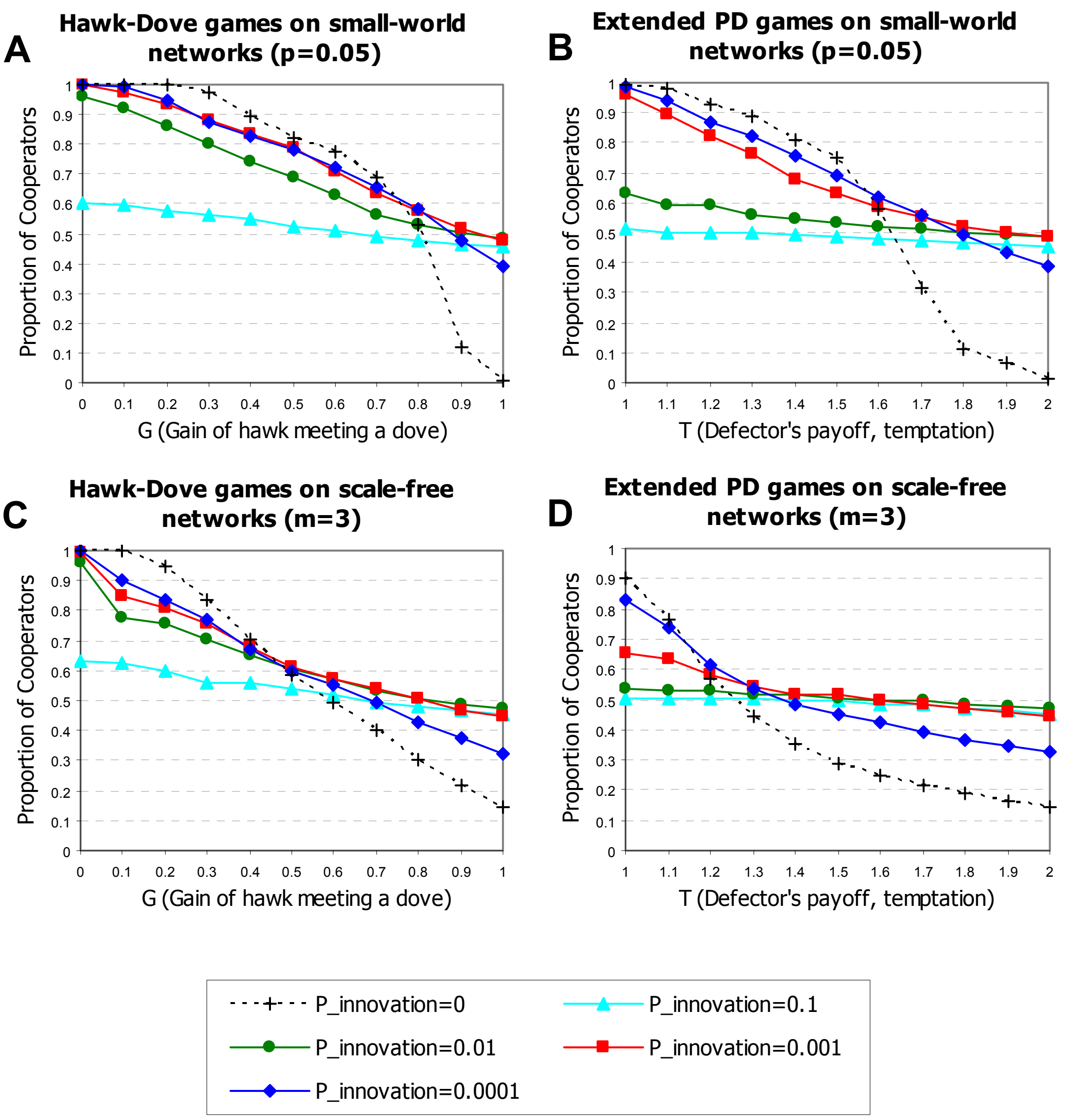

Figure S1.8. Comparison of different innovation levels of the best-takes-over long innovative strategy adoption rule in Hawk-Dove and extended Prisoner's Dilemma games on small-world and scale-free networks. The small-world (panels *A* and *B*, [2]) and scale-free (panels *C* and *D*, [10]) networks were built as described in the Methods. For the description of the Hawk-Dove game (panels *A* and *C*), extended Prisoner's Dilemma game (panels *B* and *D*) and the best-takes-over long innovative strategy adoption rule, see Methods. The probability of innovation was changed from zero to 0.1 as described in the Figure legend. For each game strategy adoption rule and *G* values (representing the gain of hawk meeting a dove, see Methods), 100 random runs of 5,000 time steps were executed.

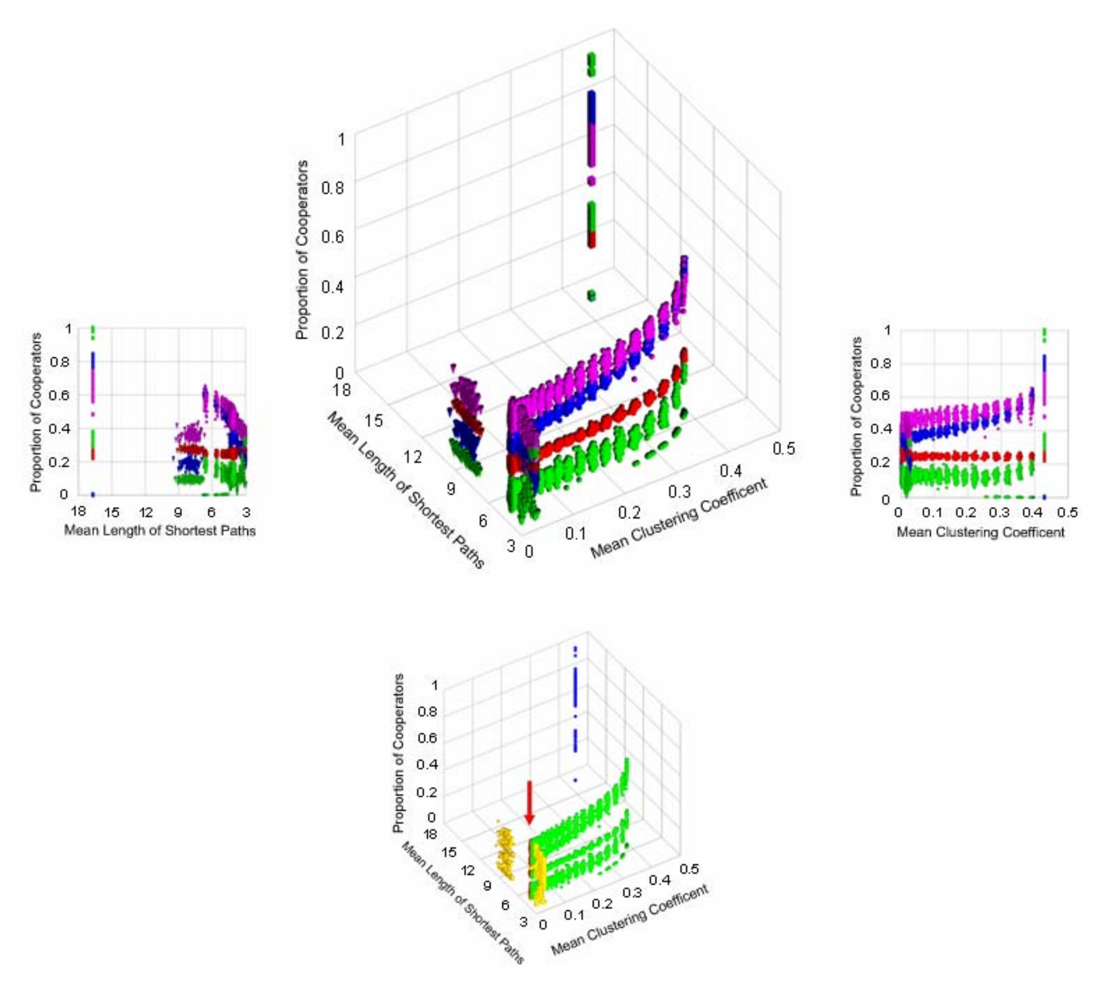

Figure S1.9. Long-term learning and innovative strategy adoption rules extend cooperative network topologies in the Hawk-Dove game. The *top middle panel* shows the level of cooperation at different network topologies. small-world (spheres) and scale-free (cones) networks were built as described in the Methods. The rewiring probability, p of small-world networks was increased from 0 to 1 with 0.05 increments, the number of edges linking each new node to former nodes in scale-free networks was varied from 1 to 7, and the means of shortest path-lengths and clustering coefficients were calculated for each network. Cubes and cylinders denote regular (p = 0) and random (p = 1.0) extremes of the small-world networks, respectively. For the description of the games and the best-takes-over (green symbols); long-term learning best-takes-over (blue symbols); long-term learning innovative best-takes-over (magenta symbols) and Q-learning (red symbols) strategy adoption rules used, see Methods. The *left and right panels* show the 2D side views of the 3D top middle panel using the same symbol-set. For each network 100 random runs of 5,000 time steps were executed at a fixed G value of 0.8. The *bottom middle panel* shows a color-coded illustration of the various network topologies used on the top middle panel. Here the same simulations are shown as on the top middle panel with a different color-code emphasizing the different network topologies. The various networks are represented by the following colors: regular networks – blue; small-world networks – green; scale-free networks – yellow; random networks – red (from the angle of the figure the random networks are behind some of the small-world networks and, therefore are highlighted with a red arrow to make there identification easier).

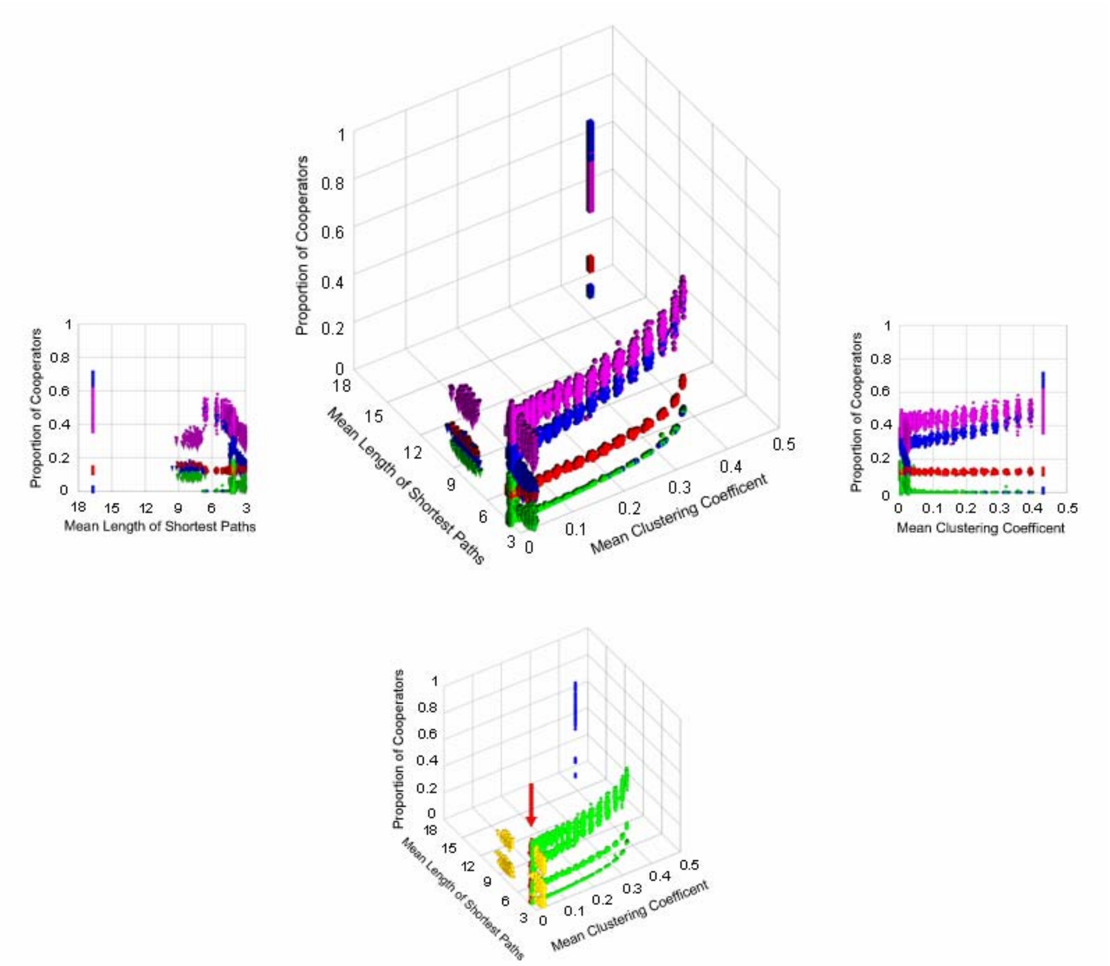

Figure S1.10. Long-term learning and innovative strategy adoption rules extend cooperative network topologies in the extended Prisoner's Dilemma game. The *top middle panel* shows the level of cooperation at different network topologies. small-world (spheres) and scale-free (cones) networks were built as described in the Methods. The rewiring probability, *p* of small-world networks was increased from 0 to 1 with 0.05 increments, the number of edges linking each new node to former nodes in scale-free networks was varied from 1 to 7, and the means of shortest path-lengths and clustering coefficients were calculated for each network. Cubes and cylinders denote regular (p = 0) and random (p = 1.0) extremes of the small-world networks, respectively. For the description of the games and the best-takes-over (green symbols); long-term learning best-takes-over (blue symbols); long-term learning innovative best-takes-over (magenta symbols) and Q-learning (red symbols) strategy adoption rules used, see Methods. The *left and right panels* show the 2D side views of the 3D top middle panel using the same symbol-set. For each network 100 random runs of 5,000 time steps were executed at a fixed *T* value of 1.8. The *bottom middle panel* shows a color-coded illustration of the various network topologies used on the top middle panel. Here the same simulations are shown as on the top middle panel with a different color-code emphasizing the different network topologies. The various networks are represented by the following colors: regular networks – blue; small-world networks – green; scale-free networks – yellow; random networks – red (from the angle of the figure the random networks are behind some of the small-world networks and, therefore are highlighted with a red arrow to make there identification easier).

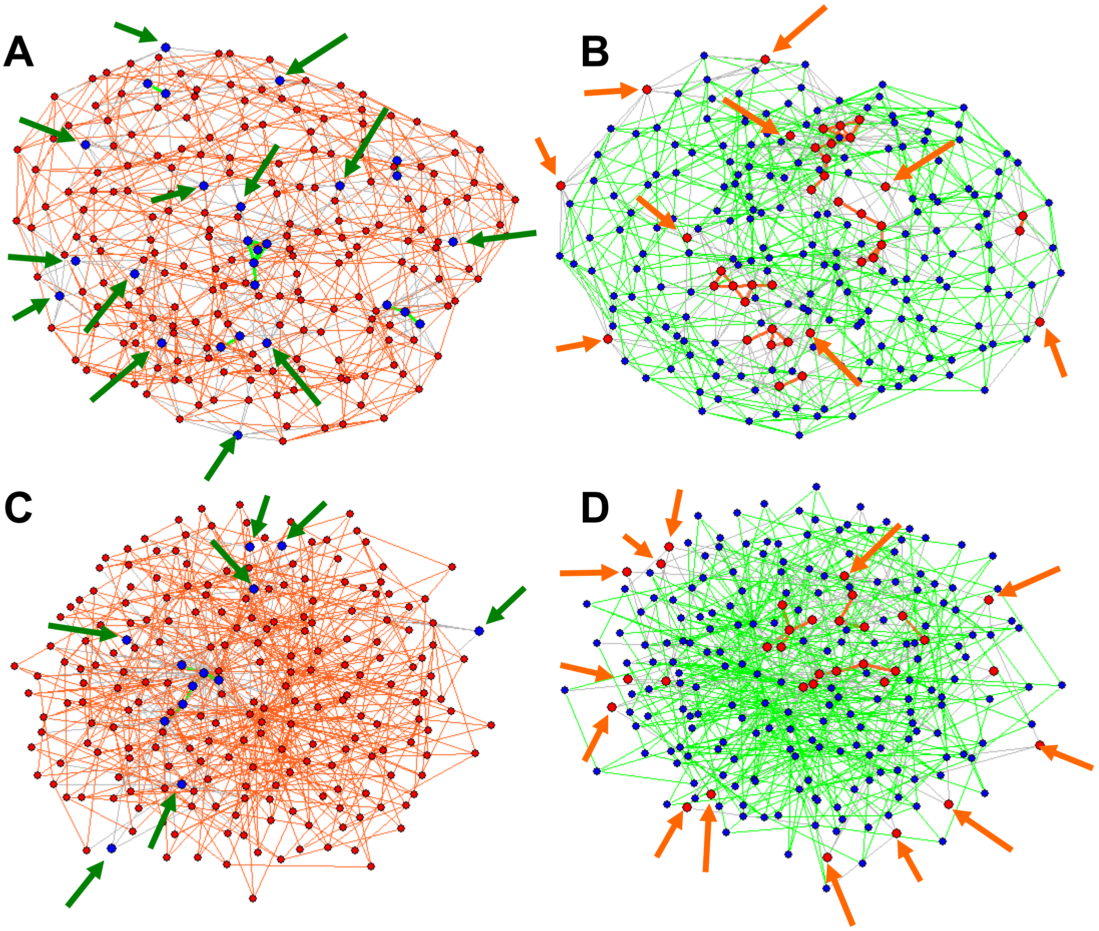

Figure S1.11. Both hawks and doves become isolated in extreme minority, when they use the innovative Q-learning strategy adoption rule in Hawk-Dove games on small-world and scale-free networks. The small-world [2] and scale-free networks [10] were built, and Hawk-Dove games were played as described in the Methods using 225 agents. Networks showing the last round of 5,000 plays were visualized using the Kamada-Kawai algorithm of the Pajek program [116]. Blue and orange dots correspond to hawks and doves, respectively. Green, orange and grey lines denote hawk-hawk, dove-dove or dove-hawk contacts, respectively. Arrows point to lonely hawks or doves using the respective colors above. *A*, Small-world network with a rewiring probability of 0.05, *G*=0.15. *B*, Small-world network with a rewiring probability of 0.05, *G*=0.95. *C*, Scale-free network with m=3, *G*=0.1. *D*, Scale-free network with m=3, *G*=0.98. We have received similar data when playing extended Prisoner's Dilemma games (data not shown).

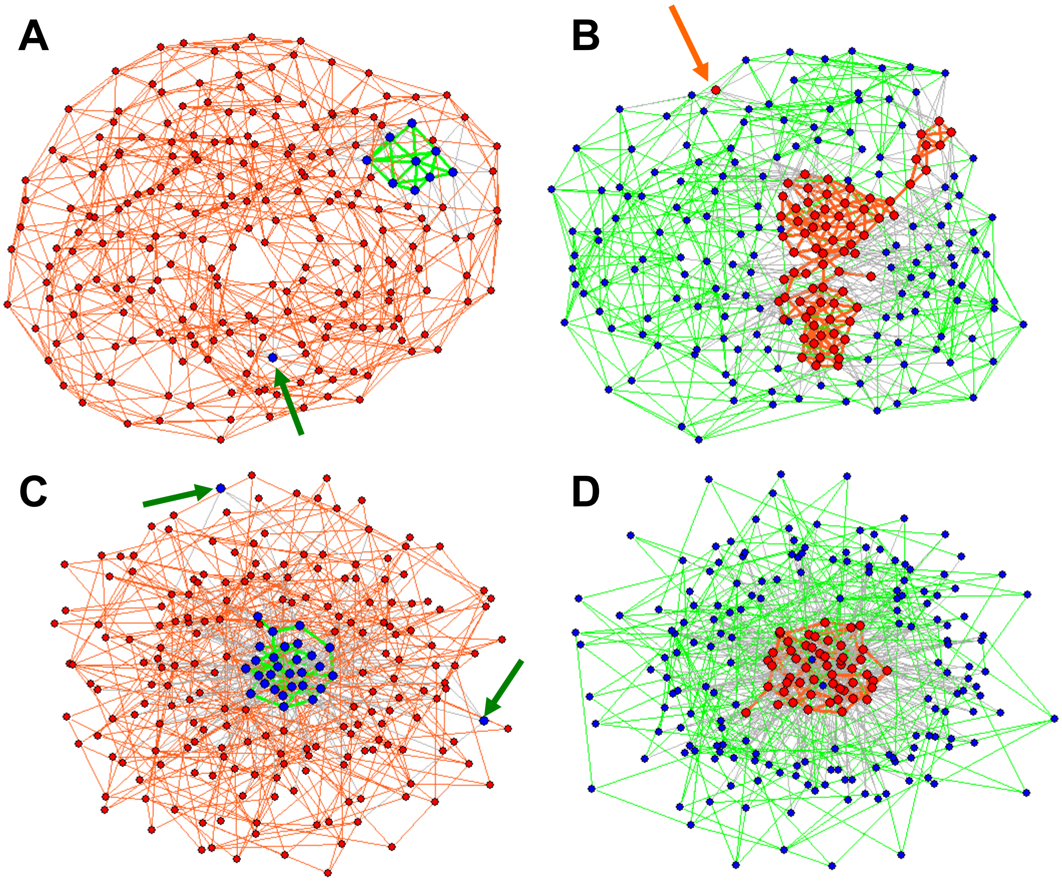

Figure S1.12. Hawks, and especially doves are not extremely isolated in extreme minority, when they use the non-innovative best-takes-over strategy adoption rule in Hawk-Dove games on small-world and scale-free networks. The small-world [2] and scale-free networks [10] were built, and Hawk-Dove games were played as described in the Methods using 225 agents. Networks showing the last round of 5,000 plays were visualized using the Kamada-Kawai algorithm of the Pajek program [116]. Blue and orange dots correspond to hawks and doves, respectively. Green, orange and grey lines denote hawk-hawk, dove-dove or dove-hawk contacts, respectively. Arrows point to lonely hawks or doves using the respective colours above. *A*, Small-world network with a rewiring probability of 0.05, *G*=0.15. *B*, Small-world network with a rewiring probability of 0.05, *G*=0.75. *C*, Scale-free network with m=3, *G*=0.1. *D*, Scale-free network with m=3, *G*=0.8. We have received similar data using other non-innovative strategy adoption rules, such as pair-wise comparison dynamics, or proportional updating, as well as when playing extended Prisoner's Dilemma games (data not shown).
